# The Potential Impact of Oral Nicotine Pouches on Public Health: A Scoping Review

**DOI:** 10.1093/ntr/ntae131

**Published:** 2024-06-17

**Authors:** Nargiz Travis, Kenneth E Warner, Maciej L Goniewicz, Hayoung Oh, Radhika Ranganathan, Rafael Meza, Jamie Hartmann-Boyce, David T Levy

**Affiliations:** Lombardi Comprehensive Cancer Center, Georgetown Medical University, Washington, DC, USA; Department of Health Management and Policy, School of Public Health, University of Michigan, Ann Arbor, MI, USA; Department of Health Behavior, Roswell Park Comprehensive Cancer Center, Buffalo, NY, USA; Lombardi Comprehensive Cancer Center, Georgetown Medical University, Washington, DC, USA; Lombardi Comprehensive Cancer Center, Georgetown Medical University, Washington, DC, USA; Department of Integrative Oncology, BC Cancer Research Institute, Vancouver, BC, Canada; School of Population and Public Health, University of British Columbia, Vancouver, BC, Canada; Department of Health Promotion and Policy, University of Massachusetts, Amherst, MA, USA; Lombardi Comprehensive Cancer Center, Georgetown Medical University, Washington, DC, USA

## Abstract

**Introduction:**

Oral nicotine pouches (ONPs) are a new class of nicotine products. This scoping review summarizes evidence on ONPs and explores their potential public health impact.

**Aims and Methods:**

We conducted a structured literature search for empirical studies across three electronic databases through January 10, 2024. Outcomes included ONP product characteristics, use patterns, beliefs and perceptions, toxicity, and marketing and sales.

**Results:**

Sixty-two studies were included, 17 were industry-funded. Most studies were from the United States. While large variations across studies were observed in ONP youth prevalence estimates, nationally representative U.S. studies find current use at 1.5% and lifetime use below 2.5% through 2023. Between 35% and 42% of U.S. adolescents and young adults have heard of ONPs, and 9-21% of tobacco-naïve youth were susceptible to trying them. U.S. adult-use estimates varied widely (0.8%–3% current; 3%–16% lifetime use) and were limited to populations with a history of tobacco use. The chemical composition of ONPs suggests fewer harmful/potentially harmful compounds at lower levels than cigarettes and smokeless tobacco (SLT), except formaldehyde. Industry-funded studies find substantially less cytotoxicity compared to cigarettes and suggest that higher nicotine-strength ONPs can deliver nicotine at levels comparable to or higher than SLT or cigarettes, although with slower nicotine release than cigarettes. Evidence on the cytotoxicity of ONPs relative to SLT is mixed.

**Conclusions:**

ONPs appear to be less toxic than cigarettes and deliver comparable nicotine, presenting an alternative for combustible product users, although key data are mainly available from industry-funded studies. Data from independent research is critically needed. Industry marketing of ONPs may encourage initiation in youth and situational and dual use in adults.

**Implications:**

The review provides an initial assessment of the potential role of ONPs in harm reduction and aims to determine unintended consequences of their use (youth uptake and dual-use) and identify populations that disproportionately use the product. This information is essential for tobacco regulatory bodies in determining the net public health impact of nicotine pouches.

## Introduction

Over the last decade, noncombustible nicotine-containing products that are potentially less harmful than combustible tobacco products have entered the global tobacco market. Harm reduction literature has since mainly focused on electronic nicotine delivery systems (ENDS)^1–4^ and heated tobacco products (HTPs).^5–8^ Recently, a new class of noncombustible nicotine products, known as oral nicotine pouches (ONPs), has emerged. ONPs are pre-portioned pouches similar in appearance and use to traditional snus (a form of smokeless tobacco [SLT] placed between gum and lip) and are sold in various flavors and nicotine strengths. Unlike SLT products that contain processed tobacco leaf, ONPs contain synthetic or tobacco-derived nicotine formulated into a white granular powder or a plant fiber-based substrate version^9^ and are often marketed as “tobacco-free.”^10^

Since their introduction to the global market after 2016, ONPs have become widely available, with the majority of sales concentrated in the United States, Sweden, and Denmark.^11,12^ Particularly in the United States, sales of ONPs continue to grow rapidly.^13^ Leading brands of ONPs are currently owned by the major tobacco companies (Table 1). With Altria buying an 80% stake in ON! in 2019 and Philip Morris International acquiring Swedish Match (ZYN) in 2022, the sales of ONPs in the United States are expected to further increase.^14^ A substantial investment in the marketing of ONPs^15^ suggests that ONPs are becoming increasingly important to the tobacco industry.

**Table 1.** Leading ONP Brands Available in the United States Between 2016 and 2022

ONP brand	Manufacturer	Nicotine strengths	Flavors	Other oral nicotine products
ZYN	Philip Morris International (Swedish Match)	3 mg; 6 mg	Cool mint, peppermint, wintergreen, spearmint, cinnamon, coffee, citrus, unflavored smooth (unflavored), menthol (unflavored), and chill (unflavored).	n/a
On!	Altria	1.5 mg; 2 mg; 3.5 mg; 4 mg; 8 mg	Mint, berry, cinnamon, citrus, coffee, wintergreen, and original (unflavored)	n/a
VELO (formerly LYFT)	British American Tobacco (BAT)	2 mg; 4 mg; 7 mg	Mint, citrus, spearmint, wintergreen, cinnamon, citrus burst, dragon fruit, coffee, peppermint, and black cherry	VELO nicotine lozenge (berry, mint, dark mint, and crema flavors; 4 mg nicotine strength)
Rogue	Swisher International	3 mg; 6 mg	Wintergreen, peppermint, apple, berry, spearmint, cinnamon, mango, honey lemon, and tabac, original.	Rogue nicotine lozenge (wintergreen, peppermint, menthol, and citrus; 2 and 4 mg nicotine strength) Rogue nicotine gum (wintergreen, peppermint, fruit; 2 and 4 mg nicotine strength) Rogue nicotine tablet (wintergreen, peppermint, berry; 2 and 4 mg nicotine strength)

In the United States, the potential public health impact of ONPs is especially relevant because of steadily rising trends in awareness and use, particularly among young adults.^16,17^ As with ENDS,^18,19^ the wide variety of ONP flavors and aggressive marketing campaigns (eg, advertising ONPs as “flavor-ban approved,” using social media campaigns)^20–22^ have the potential to appeal to youth and young adults, providing another pathway to nicotine dependence. However, because ONPs do not involve combustion or contain tobacco leaves, they may be a lower-risk product if used as a substitute to replace more harmful types of tobacco, especially combustibles.^23^

Classification and regulation of ONPs vary substantially across countries. Some classify ONPs as nicotine replacement therapies regulated as medicinal products (eg, Finland, Spain, and Australia [for synthetic nicotine ONPs]), and others classify them as tobacco products (eg, Brazil and United States).^11^ In other countries, ONP sales are banned or proposed to be banned (eg, Netherlands, Belgium, and Singapore) or are unregulated (eg, Germany, Sweden, and United Kingdom).^11,24,25^ In the United States, the Food and Drug Administration (FDA) has asserted jurisdiction over ONPs, but no ONP product has yet received premarketing authorization. Hence, ONPs do not neatly fit into pre-established product categories and present a new challenge to regulators seeking to shape markets and protect public health.

Conceptual frameworks and regulatory agendas have been proposed to help inform regulatory actions.^23,26^ However, due to the product’s novelty, empirical evidence is needed to assess the potential role of ONPs in harm reduction and to identify unintended consequences of their use (eg, concurrent use with other tobacco products and youth uptake).

This scoping review summarizes the literature on ONP product characteristics, toxicity, use prevalence estimates, product appeal, risk perceptions, sales trends, and industry marketing tactics. We aim to provide direction for future research on ONPs useful to tobacco regulatory bodies.

## Materials and Methods

We used a scoping review methodology to answer a broad research question of what is known about ONPs and to summarize findings from existing research, which we judged likely to be heterogeneous in study designs, methods, and outcomes. We adhered to guidelines for the Preferred Reporting Items for Scoping Reviews (PRISMA-ScR).^27^

### Search Strategy

Following the review protocol, two reviewers (NT, HO) carried out a structured literature search in PubMed (MEDLINE), Web of Science, and Embase databases through January 10, 2024. We limited the searches to 2016 onwards, when ONPs first emerged in the U.S. market. The complete search strategy employed for PubMed is presented in File S1.

Two reviewers (NT and HO) independently extracted and screened titles and abstracts using the Distiller SR Literature Review Software (Version 2.35; DistillerSR Inc.; 2023). Reviewers were blinded to author names, affiliations, and publication journals. Two reviewers (NT and HO) independently screened full-text articles for eligibility criteria. Finally, two reviewers (NT and RR) searched reference lists in the bibliographies of eligible papers for additional sources. Disagreements between reviewers were resolved by consensus.

### Eligibility

Eligible articles had to investigate ONPs in relation to any of five outcome categories: (1) Use Patterns (prevalence estimates, sociodemographic characteristics of users, and reasons for use); (2) Beliefs and Perceptions (product awareness, appeal, satisfaction, and risk perception); (3) Product Characteristics (nicotine content and pharmacokinetics); (4) Toxicity (toxicant content, in vitro/in vivo toxicity, and human health outcomes); and (5) Marketing and Sales (sales analysis, industry marketing strategies, advertising expenditures, and retail availability).

We included empirical studies of any design published in English and excluded reviews, editorials, commentaries, and conference proceedings. Studies that investigated oral nicotine products (eg, nicotine pouches, lozenges, and gum) as a category, without reporting nicotine pouch-specific findings, were excluded.

### Data Extraction

Two reviewers (NT and HO) independently extracted data from eligible studies using an a priori-developed standardized data extraction tool in Microsoft Excel (Version 2310; Microsoft Corporation; 2019). Information was extracted for citation, funding, author financial disclosures, study design, data collection period, country of investigation, product definition, product characteristics (flavor, nicotine strength, and brand), comparator product, population characteristics (age, socioeconomic status, ethnicity/race, and history of tobacco or nicotine product use), description of intervention/exposure, cell-type (for in vitro studies), main results, authors’ conclusions, and study limitations.

### Data Synthesis

Our primary analysis consists of a detailed narrative and tabular presentation of the findings grouped by outcome category. The secondary analyses stratify findings by population, document differences in findings between industry versus non-industry-funded studies, and identify gaps and limitations of current research.

## Results

**Figure 1.**
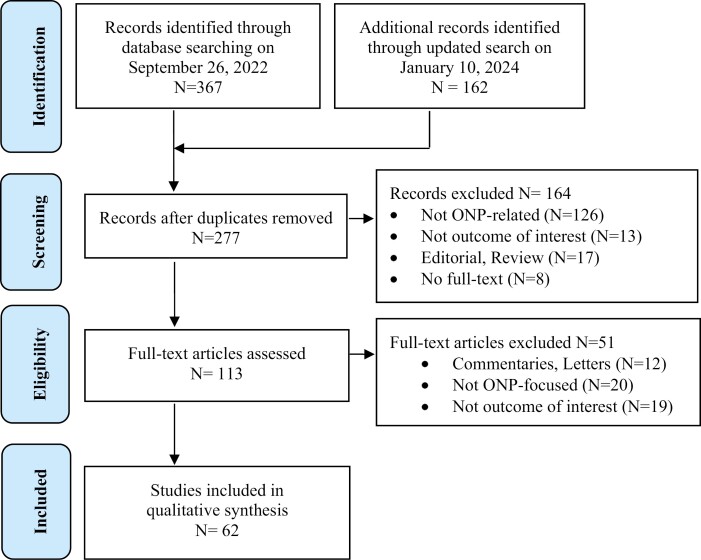
PRISMA Flow-chart of eligibility screening.

A database search identified 277 unique records, of which 113 were further assessed for eligibility in full text. A total of 62 studies were eligible for inclusion, with 17 funded by industry (Figure 1). Most studies were from the United States (43), with some from Sweden (6), United Kingdom (4), Canada (4), Germany (3), the Netherlands (1), and Australia (1). Table S1 summarizes the characteristics of all included studies. The age ranges of the surveyed populations varied across studies and are presented in Tables S1–S3.

### Use Patterns of ONPs

#### Prevalence Estimates of ONP Use

Estimates of current (mostly past 30 days) and lifetime (ever) use among youth, young adults, and adults in the United States and other countries are presented in Table S2.

##### United States

Data from nationally representative and population-based youth surveys show lifetime ONP use among adolescents at 3.5%–4.1 % between 2019 and 2021,^28^ at 1.9% in 2021^29,30^ and at 2.3% in 2022^31^ and 2023.^32^ Current use was reported by 1.5%–2.0% of adolescents between 2019 and 2021,^28,33^ 0.8% in 2021,^29,30^ 1.1% in 2022,^31^ and 1.5% in 2023.^32^ Non-representative surveys report from 0.6%^34^ lifetime use in adolescents to 16%–18%^35,36^ lifetime and 11%–12% current use^35,36^ in youth and young adults in 2021–2022, with higher estimates of lifetime (11%) and current (29%) ONP use among ENDS users.^37^

Adult prevalence estimates from two 2021 nationally representative surveys are limited to those with a history of tobacco use and estimate current use at 3% among current and former tobacco users,^38^ and lifetime use at 5.6% among current smokers^16^ and 16.4% among current and former tobacco users.^38^ Various non-representative surveys estimated current use below 1% among current and former tobacco users in 2020^17,39^ and at 2.2% among young adult cigarette and ENDS users in 2022.^40^ Lifetime ONP use was reported at 3% in a sample of current and former adult smokers and ENDS users in 2020^17^ and at 10% among young adult current tobacco users in 2021–2022.^40–42^

##### Other Countries

Lifetime ONP use among adolescents was reported at 0.3% and current use at 0% in the Netherlands,^43^ while current use in Canada^33^ and England^33^ was below 1.5% in 2019–2020 nationally representative surveys. Among adults, current ONP use was below 0.5% in the Netherlands^43^ and Great Britain^44^ in 2020–2021, while nearly 3% of current and former smokers reported current ONP use in the United Kingdom in 2019.^45^

#### Demographic Characteristics of ONP Users

Studies investigating demographic characteristics of lifetime ONP users were largely heterogeneous in their samples and focused mainly on adult current and/or former tobacco product users (Table S3). Except for two UK studies,^44,45^ all were from the United States. The majority of studies found a higher likelihood of lifetime ONP use for younger adults^17,35,41^ or those ages 18–45,^16,38,45^ males,^17,35,42,45^ current^17,38^ and lifetime^16,41,42^ SLT users, and current cigarette and ENDS users.^45^ Lifetime cigarette, cigar, and ENDS use were not associated with lifetime ONP use,^16,42^ except in one survey.^41^ The findings for lifetime ONP use and ethnicity, education, and income were inconsistent across studies: Positively associated with being white and higher education levels,^17,42,45^ not associated with ethnicity and income,^16,17^ and positively associated with lower levels of education^16^ and lower income.^35^ Among U.S. adolescents, higher lifetime ONP use was observed in male and non-Hispanic white populations,^28–32^ and those reporting past 30-day SLT, ENDS, and cigarette use.^28^

Demographic characteristics of current ONP users were less studied due to small sample sizes. Studies suggest that current ONP users are likely to be young adults^35,38^ and ages 35–44,^38,44^ males,^35,44,46^ and current SLT,^38,46^ cigarette,^38,44^ and ENDS^38,44^ users as well as former smokers.^44,46^

#### Reasons for ONP Use

##### Non-Industry-Funded Studies

A U.S. survey^40^ of youth and young adults ages 18–34, many of whom used cigarettes and ENDS, found curiosity about the product (28%) and flavors (26%), use where other tobacco products are prohibited (26%), and discreet use (29%) to be the strongest motives for ever ONP use. In a Dutch survey^43^ of adolescents and adults ages ≥13 most common reasons for ever ONP use were curiosity about the product (over 70%), and perceived reduced harm compared to cigarettes (over 20%). The availability of flavors (31%) was the main motive for use in a U.S. sample of ONP-experienced adults.^47^

##### Industry-Funded Studies

A Swedish Match-funded U.S. study^46^ found that less perceived harm compared to cigarettes was the most common reason for ZYN use among current cigarette smokers (73% of respondents) and dual cigarette and SLT users (60%) who started using ZYN. The less perceived harm compared to other tobacco products was the predominant reason for using ZYN among current SLT users (65%), current other tobacco product users (71%), former tobacco users (63%), and never-tobacco users (51%). Ease of pouch use (53%) and helping to reduce (46%) or quit (52%) other tobacco use were among other common reasons.

### Beliefs and Perceptions

#### Product Awareness

##### US Youth and Young Adults

Nearly 36% of middle and high school students had heard of ONPs in a 2021 representative survey.^29^ Awareness was especially prevalent among ever and current users of SLT, ENDS, cigarettes, cigars, and HTPs. A 2021 Southern California survey^48^ of young adult never-ONP users ages 19–23 found that nearly 11% were aware of ONP products, with greater awareness in current noncombustible product users (19%) and dual (combustible and noncombustible) users (20%). A 2021 survey^41^ of young adults ages 18–25 (with current tobacco users oversampled) found nearly 42% awareness of ONPs. In another 2021 survey, 37% of young adults ages 18–25 were aware of synthetic nicotine ONPs, in particular.^42^ Higher odds of awareness were associated with younger age and lifetime cigarette and cigar/cigarillo use. A 2022 survey^40^ of young adults ages 18–34 (with oversampled cigarette and ENDS users) found 35% ONP awareness, with higher odds among males, nonwhite participants, and those using cigarettes, ENDS, and SLT.

##### US Adults

A 2020 survey^17^ found that nearly 20% of adults with a history of smoking and/or vaping had heard of ONPs, with increased awareness among younger ages 18–24 and those ages 40–54 compared to ≥55, and among current exclusive ENDS users, dual (ENDS and cigarette) users, and SLT users compared to non-current users. Representative surveys found that in 2021, nearly 29% of adult current established smokers^16^ and nearly 47% of current and former adult tobacco users^38^ were aware of ONPs. Higher awareness was observed for ages 18–29 and 30–44 compared to ages >60, and by ever SLT user.^16^

##### Other Countries

In a 2019 UK representative survey,^45^ 16% of adults with a smoking and/or vaping history had heard of ONPs. In a 2020 representative survey^43^ of Dutch adolescents and adults, 7% were aware of ONPs, with higher awareness among adolescents (9%) and young adults ages 18–24 (10%) and 25–44 (9%) compared to ages > 44.

#### Product Interest/Susceptibility

##### US Youth and Young Adults

Measures of product susceptibility varied, but generally included not being opposed to trying ONPs. Among Southern California adolescent never-tobacco users in 2021, between 9%^49^ and 21%^50^ were susceptible to trying ONPs after viewing product images. A 2021 Southern California survey^48^ of young adult never users of ONPs ages 19–23 found 19% susceptible to trying ONPs after viewing ONP product images and advertising. Susceptibility was lower among non-users of any tobacco products (15%) compared to combustible product users (29%), exclusive noncombustible product (snus, ENDS, or HTP) users (34%), and multi-product users (44%). In a 2021 survey^42^ of young adults ages 18–25, 29% were susceptible to trying synthetic nicotine ONPs in the next year, with higher odds in males, and in lifetime cigarette, ENDS, and SLT users compared to non-users. Nearly 24% of young adults ages 18–25 were susceptible to ONP use in a 2021 survey,^41^ with current tobacco users oversampled.

##### US Adults

In a 2021 representative survey,^38^ 43% of adult current and recent former tobacco users were susceptible to ONP use. Another 2021 representative survey^16^ had nearly 17% of adult current established smokers expressing interest in using ONPs in the next 6 months, with greater interest observed in those who planned to quit smoking in the next 6 months or who previously tried quitting smoking, and those who ever used ONPs.

##### Industry-Funded Studies

In a Swedish Match-funded adult consumer panel,^46^ 75% of dual SLT and cigarette users found ZYN appealing after viewing its packaging and product description, followed by 52% of current SLT users, 36% of current smokers, 12% of former tobacco users and 11% of never-tobacco/nicotine users.

#### Risk Perception

In a Southern California survey^48^ of young adult never users of ONPs nearly half were uncertain whether ONPs posed less harm to health than cigarettes (49%) and ENDS (52%). Uncertainty about the harm of ONPs relative to traditional SLT was also reported in a U.S. sample^41^ of young adults (with current tobacco users oversampled). In a U.S. representative sample^38^ of adult current and former tobacco users, 23% agreed that ONPs were less harmful than SLT, while 40% disagreed and 37% were unsure. ONPs were generally rated less harmful compared to cigarettes, cigarillos, and HTPs in a representative sample of Dutch adolescents and adults.^43^ Susceptibility to ONP use and current and lifetime ONP use were associated with favorable harm perceptions of ONPs compared to SLT and other tobacco products.^38,40,41^ In particular, awareness of and susceptibility to synthetic nicotine ONP use in young adults was associated with their less perceived harm than tobacco-derived ONPs.^42^

Experimental studies show that ONP product packaging influences risk perception. Viewing ONP packaging with a “tobacco-free” warning label was associated with reduced harm perception in young adult men^51^ and with reduced risk perceptions and increased use intentions among youth, non-tobacco users, and racially minoritized groups^52^ compared to viewing a standard FDA nicotine warning label. Risk perceptions also varied by tobacco use behavior. Viewing ONP packaging with a modified-risk tobacco product warning label was associated with reduced harm perception compared to cigarettes, but not ENDS in young adult cigarette/ENDS users.^53^ Adult current smokers perceived ONPs to have similar overall health risks to cigarettes (less respiratory, but more oral and gastrointestinal), while SLT users viewed ONPs as similarly or less risky than SLT.^54^ ONPs were perceived as less harmful by adult tobacco users versus non-tobacco users after viewing ONP pack images, regardless of the warning label presence.^55^

#### Subjective Ratings

Five industry-funded and one non-industry-funded randomized crossover studies examined subjective ratings of ONP use in established adult tobacco users, including product likability, satisfaction, intent to use again, changes in urges to smoke, and cravings for a cigarette.

##### Non-Industry Funded Studies

In one study,^56^ smokers found ONPs to be moderately appealing, but less appealing than usual brand cigarettes. Initial withdrawal relief was greater post-cigarette use, but remained comparable for both products during a 90-minute follow-up.

##### Industry-Funded Studies

In a BAT-funded study,^57^ satisfaction with different brand ONPs was assessed in current dual snus and cigarette users after a single cigarette or ONP product use. Higher scores for product likability were observed for combustible cigarettes (60%), followed by ZYN 10 mg (Swedish Match, 54%), and lowest for the lowest strength nicotine ONP (Altria’s ON! 6 mg, 29%). Higher scores for intent to continue to use the product were received for combustible cigarettes (57%) than all ONPs (14% for ON! 6 mg- 37% for ZYN 10 mg). In an Altria-funded study^58^ of smokers randomly assigned to ONP or own-brand cigarette use, the desire to use the product again was highest for cigarettes (86%), with ratings also high for six favors of ON! 4 mg pouches (71%–79%). Reduction in urges to smoke, cigarette craving, and positive subjective ratings (eg, pleasant, satisfying, and calming effects) were lower after ONP use of all flavors compared to cigarette use. In another Altria-funded study,^59^ subjective ratings of ON! were similar between 1.5 and 8.0 mg nicotine strengths, but lower compared to participants’ own-brand cigarettes and moist SLT in dual cigarette and moist SLT users. The reduction in cigarette craving and urges to smoke post-product use was comparable between ON! 8 mg and cigarettes and moist SLT. The desire to use the product again was highest for moist SLT (93%) and cigarettes (77%) compared to ONPs (46% for 8.0 mg—66% for 1.5 mg). In an Imperial Brands study,^60^ product likability among tobacco users was similar for cigarettes compared to 5.8 and 10.1 mg ZoneX pouches. In an Imperial Tobacco Canada-funded study^61^ of cigarette smokers, a 4 mg ONP showed greater product likability with 55% of responses compared to a 4 mg nicotine lozenge (19%) and 4 mg nicotine gum (9%). Product satisfaction was highest for nicotine gum (50% of responses), followed by ONP (40%) and lozenge (16%).

Overall findings indicate greater product likability, satisfaction, and desire for repeat use of combustible cigarettes and SLT than ONPs in adult smokers and dual cigarette and SLT users.

### Product Characteristics

#### Nicotine Content and Release From Pouches

Four studies analyzed the nicotine content in ONPs, with two funded by industry

##### Non-Industry-Funded Studies

An analysis^62^ of 37 ONPs of different brands, nicotine strengths, and flavors yielded a range of total nicotine content from 0.89 mg/pouch (Velo 2 mg) to 6.73 mg/pouch (White Fox). ON! and White Fox exhibited the highest alkalinity levels (pH of 9.36), corresponding to 95.8% free-base nicotine in ON! 3 mg, 97.3% in ON! 6 mg, and 99.2% in White Fox pouches. Higher alkalinity (pH > 6) increased the amount of bioavailable free-base nicotine (the form most easily absorbed), suggesting nicotine delivery properties of ONPs comparable to snus. In another study,^63^ the nicotine content of 44 ONPs from 20 brands available in Germany ranged from 1.79 to 47.5 mg/pouch, with a median value of 9.48 mg/pouch. The pH levels ranged from 5.5 to 10.5, with a median of 8.8, while the median proportion of free-base nicotine was 86%.

##### Industry-Funded Studies

In a Swedish Match study,^64^ the amount of in vivo extracted nicotine from ZYN 6 mg (3.51 mg/pouch) after a 60-minute application by a sample of snus users was higher than from their General Snus 8 mg (2.41 mg/sachet). ZYN 8 mg yielded a higher amount of extracted nicotine (3.79 mg/pouch) than Longhorn 18 mg moist snus (2.99 mg/sachet) but lower than two 8mg General Snus sachets (5.04 mg). The extracted fraction of nicotine was higher from 3 mg (56%) and 6 mg (59%) ZYN than from General Snus 8 mg (32%) and higher from ZYN 8 mg (50%) than from both reference products (19% for Longhorn snus and 33% for two 8 mg General Snus sachets). A study^61^ by Imperial Tobacco Canada found that the fraction of extracted nicotine from a pouch after a 60-minute application varied substantially within a sample of smokers, with mean values higher for the 4 mg ONP (62%) than the 4 mg nicotine gum (33%), but lower than the 4 mg nicotine lozenge (100%).

In summary, both industry and non-industry studies indicate levels of total and free-base nicotine in ONPs comparable to conventional SLT, suggesting the ability to deliver nicotine at a similar concentration as SLT. However, studies also indicate a high degree of variability in nicotine content between ONP brands.

#### Pharmacokinetics

All but one randomized crossover study analyzing the pharmacokinetic properties of ONPs were funded by industry (Table S4).

##### Non-Industry Funded Study

In adult smokers, using ZYN 6 mg was associated with greater plasma nicotine delivery at 30 minutes than ZYN 3 mg or cigarettes.^56^

##### Industry-Funded Studies

The nicotine plasma concentration after using a ZYN 6 mg pouch was higher than General Snus 8 mg in snus users, while for ZYN 8 mg the concentration was comparable to Longhorn 18 mg moist snuff.^64^ An analysis of six flavors of ON! 4 mg suggested slower nicotine release and lower nicotine plasma concentration compared to smokers’ own cigarette brands.^58^ A subsequent analysis^59^ of various nicotine strength ON! pouches found lower plasma concentrations for lower strength ONPs (<4 mg) compared to own-brand cigarette and moist SLT in dual cigarette and moist SLT users, while the pharmacokinetic profile of ON! 4 mg was comparable to moist SLT, and the profile of ON! 8 mg substantially exceeded that of cigarettes and moist SLT. Nicotine release was slower for all ONPs than for cigarettes, and comparable to moist SLT.

The nicotine plasma concentration of LYFT 10 mg was substantially greater than for a cigarette and other ONPs (ZYN 10 mg and Skruf 8 mg) in dual snus and cigarette users, while nicotine release was slower for all ONPs than for a cigarette.^57^ Nicotine delivery was lower and slower for both 5.8 and 10.1 mg ZoneX ONPs compared to cigarette in cigarette smokers or snus users.^60^ Nicotine plasma concentration of BAT-manufactured 4 mg ONPs in smokers were similar to those of the same strength nicotine lozenge, but much higher than those of nicotine gum.^61^

Overall, studies suggest a lower plasma nicotine concentration for lower-strength ONPs (<4 mg) compared to cigarettes and SLT, while higher-strength ONPs (≥6 mg) may deliver comparable or higher nicotine than conventional SLT products and cigarettes.

### Toxicity

#### Toxicant Content

Evidence on ONP constituents was obtained from five chemical studies, two were industry-funded.

##### Non-Industry-Funded Studies

A screening^65^ of 48 ONPs from 22 manufacturers showed 186 different chemical components present besides nicotine, with an average of 17 constituents in each ONP. Eight were classified as hazardous according to the European Classification Labeling and Packaging regulation, and methyl eugenol, benzophenone, and β-myrcene were classified as possibly carcinogenic to humans by the International Agency for Research on Cancer. Among other carcinogens, tobacco-specific nitrosamines (TSNAs) were detected in 26 of the 44 ONP samples from 20 manufacturers,^63^ although often below quantification levels. The authors report that the highest detected levels were much lower than those typically found in cigarettes and snus.

In an analysis^9^ of 21 white granular powder-based or plant fiber-based ONPs, levels of benzo[a]pyrene, nitrite, acetaldehyde, and metals were generally found to be either below quantification levels or at far lower levels compared to snus products, with some notable variations between samples. For example, in some plant-based ONP samples, levels of acetaldehyde, nitrite, and nickel were substantially higher than in snus products. Quantifiable TSNA levels were observed in two plant-based ONP samples, but at much lower levels than in snus. Formaldehyde was consistently found in all ONPs at levels comparable to snus, except in two white granular powder ONPs with concentrations three-to-four times higher than in snus products.

##### Industry-Funded Studies

A Swedish Match-funded analysis^66^ quantified the fewest non-nicotine compounds in the NRT lozenge (nickel), followed by ZYN moist (formaldehyde, chromium, ammonia), ZYN dry (formaldehyde, chromium, ammonia, and nickel), and NRT gum (cadmium, chromium, lead, nickel, acetaldehyde, ammonia, and the uranium isotopes). Both ZYN and NRT products contained the lowest levels of detected compounds. Snus and moist snuff contained the highest number of compounds (19 and 26, respectively), with the highest levels observed for moist snuff. While ZYN moist and snus products contained comparable levels of formaldehyde, ZYN dry exceeded those levels by five times. Notably, the study detected no TSNAs in ZYN products.

Apart from moisture and nicotine, only chromium and formaldehyde were detected in some LYFT ONP samples in a BAT-funded study.^67^ TSNA levels were below quantification. Chromium and nickel were detected in one of the three replicates of the comparator nicotine lozenge. Cadmium, chromium, nickel, and lead were present at low levels in the nicotine gum. In contrast, Swedish snus products contained 11 chemical compounds. Notably, levels of formaldehyde in ONPs were higher than in both NRT products and comparable to Swedish snus. Table S5 shows how highest detected levels of common toxicants found in ONPs compared to reference products.

Overall, both industry and non-industry-funded studies consistently found formaldehyde in ONPs, sometimes at levels much higher than in reference snus. Chromium, ammonia, and nickel were also detected in many ONP samples. TSNAs were quantifiable in some samples in two non-industry-funded studies, but not in industry-funded studies.

#### In Vitro *Toxicity*

Five out of eight studies assessing the in vitro toxicity of ONPs were industry-funded (Table 2).

**Table 2.** In Vitro Toxicity of Oral Nicotine Pouches Compared to Other Tobacco Products

Author, year	Funding	Test product	Toxicity: ONP	Toxicity: Reference product 1	Toxicity: Reference product 2
Bishop et al., 2020^68^	BAT	LYFT, Berry Frost 4 mg	Minimal cytotoxicity in all assays	*Snus* Minimal to moderate cytotoxicity in all assays	*Cigarette* Consistent toxicological effects in most assays
East et al., 2021^69^	BAT	LYFT, 4–11 mg, various flavors	All deemed non-cytotoxic (below the 30% toxicity threshold)	*Competitor ONP* (Nordic Spirit) 86% toxicity	*Snus* 91% toxicity
Yu et al., 2022^70^	Imperial Brands	Skruf Superwhite (mint, 5.8 and 10.1 mg)	Minimal cytotoxicity (above the 20% toxicity threshold)	*Snus* Minimal to moderate cytotoxicity (above the 20% toxicity threshold)	*Cigarette* Substantially more cytotoxic
Alizadehgharib et al., 2022^71^	Swedish Match	ZYN (cinnamon, smooth, peppermint, 3 or 6 mg)	No inflammatory response	*Competitor ONP* (LYFT Ice Cool) Significant increase in the production of proinflammatory cytokines	*Four snus products* Significant increase in the production of proinflammatory cytokines
Shaikh et al., 2022^72^	U.S. National Institutes of Health	Multiple nicotine strengths of different flavor ONPs (ZYN, ON!, VELO, and Rogue)	Increased cytotoxicity and inflammatory response compared to non-treated cells	*Snus* Cytotoxic effects of ZYN spearmint were comparable to those of identically flavored reference snus product.	
Shaikh et al., 2022^73^	U.S. National Institutes of Health	ZYN Smooth 6 mg, Lucy Spearmint 8 mg, ON! 8 mg Citrus	Increased cytotoxic response for ON! (20%–34%) and Lucy (13%–19%) at all concentrations, and ZYN (15%–30%) at higher concentrations.	*Snus* Increased cytotoxic response of 13%–16% at higher concentrations for snus #1, and 21%–27% at all concentrations for snus #2.	
Miller-Holt et al., 2022^74^	JTI	Nordic spirit spearmint and mint of multiple strengths	non-mutagenic, non-genotoxic and non-cytotoxic	*Snus* non-mutagenic, non-genotoxic and non-cytotoxic	*Cigarettes* mutagenic, genotoxic, and cytotoxic
Rinaldi et al., 2023^75^	Federal	Five ONPs of various flavors and nicotine strengths	Cytotoxicity was observed in two of the five ONPs.	*Snus* No cytotoxicity	

BAT = British American Tobacco; JTI = Japan Tobacco International.

##### Non-Industry-Funded Studies

Comparative analyses^72,73^ of inflammatory response, reactive oxygen species (ROS) production, and cytotoxicity between identical flavors of four various nicotine strengths of major ONP brands found increased cytotoxicity, differential ROS, and proinflammatory cytokine release in human bronchial epithelial cells compared to untreated cells at lowest concentration treatments at 4–24 hours. In particular, cytotoxic effects of ZYN 6 mg spearmint were comparable to those of identically flavored reference snus products.^72^ In human oral gingival epithelial cells, increased cytotoxicity, differential ROS, and cytokine release were observed in ONPs at the highest nicotine concentration, particularly for tobacco-, citrus-, and cool cider-flavored pouches.^73^ ONPs were found to induce an oxidative stress response rather than an inflammatory response in human gingival fibroblasts after 24 hours of exposure, compared to snus that induced both.^75^ While the referenced snus product did not exhibit any cytotoxicity, two of the five ONP samples did. The authors concluded that flavorings likely contributed to the toxicity of ONPs.^75^

##### Industry-Funded Studies

A BAT-funded study found LYFT 4 mg to be minimally toxic in all cytotoxicity assays, whereas snus showed minimal to moderate, and cigarettes showed consistent toxicological effects in most assays of human oral fibroblasts and human lung epithelial cells.^68^ In a subsequent BAT study,^69^ all LYFT ONPs (4–11 mg) were deemed non-cytotoxic in a short-term assay using human bronchial epithelial and human gingival fibroblast cells. In contrast, the competitor ONP (Nordic Spirit, JTI) and the reference snus induced up to 86%–91% cytotoxicity. Flavor and nicotine strength did not affect the overall toxicity of the ONPs.

In a JTI-funded study, Nordic Spirit ONPs of various nicotine strengths and the reference Swedish snus were found non-mutagenic, non-genotoxic, and non-cytotoxic in Chinese hamster ovary cells compared to the reference cigarette.^74^ A study by Swedish Match showed that exposure of human peripheral blood mononuclear cells to ZYN (3 and 6 mg) did not elicit an in vitro inflammatory response, while four Swedish snus products and the competitor ONP (LYFT and BAT) exhibited a significant increase in proinflammatory cytokine release compared to unstimulated cells.^71^ An Imperial Brands-funded study showed that their Skruf Superwhite ONPs (5.8 and 10.1 mg) and a Swedish snus reference product of identical flavor were deemed cytotoxic in human lung epithelial and liver cancer cell lines, although substantially less cytotoxic than the reference cigarette.^70^ Compared to a cigarette, neither ONPs nor the reference snus were found to be mutagenetic or genotoxic.

Overall, non-industry-funded studies are scarce and suggest cytotoxic effects of ONPs comparable to or exceeding those of SLT in some samples. Industry-funded findings suggest that ONPs are minimally cytotoxic, likely less cytotoxic than SLT, and substantially less cytotoxic than cigarettes. Variations in findings were observed between ONP brands. While own-brand products were typically reported as non-cytotoxic or minimally cytotoxic, the same brand products were found to be substantially more cytotoxic and comparable to snus when used as a reference product in another study, often funded by a competitor brand.

#### Human Studies

All four studies examining the human health effects of ONPs were industry-funded. The almost complete substitution of Swedish snus with 3 or 6 mg ZYN during a 6-week period resulted in the gradual resolution of preexisting oral mucosal lesions in healthy snus users.^71^ Exposure to LYFT 10 mg for 86 days resulted in minimal tooth enamel staining compared to cigarette smoke and snus.^76^ Compared to smokers, urine and blood samples of exclusive VELO users showed lower levels of biomarkers of exposure and potential harm, except for one biomarker of oxidative damage and two biomarkers related to cardiovascular disease incidence that were not significantly different from those of smokers.^77^ Completely switching from smoking to either 2, 4, or 8 mg ON! use for 7 days reduced levels of biomarkers of exposure to tobacco-related toxicants (comparable to stopping all tobacco use) in urine and blood samples of smokers relative to those who continued smoking.^78^

### Marketing and Sales

#### Market Share and Sales Data

Sales data from the 2011–2019 Nielsen Convenience Track system^79^ indicate that oral nicotine product sales steadily increased to constitute 4% of the overall U.S. SLT market by 2019, with the vast majority of sales attributed to ONPs. The most sold ONP flavors were spearmint/mint (55%) and wintergreen (23%). By 2019, the U.S. ONP market leader was ZYN, capturing 86% of the ONP market.^79^ An analysis^80^ of U.S. Nielsen sales data showed that ONP sales increased from 163 178 units in 2016 to 45.97 million units in 2020, with ZYN comprising nearly 79% of ONP market share in 2020, followed by ON! (10.0%), VELO (7.6%) and Rogue (2.4%). A subsequent Nielsen sales data analysis^13^ showed a further sales increase to 808.14 million units by 2022, with ZYN leading the overall unit share (58.8%), followed by ON! (24.6%), VELO (12.1%), and Rogue (4.8%).

#### Advertising Expenditures and Industry Marketing Strategies

While conventional SLT products accounted for 63% of total US smokeless tobacco advertising expenditures between 2018 and 2020, ONP advertising exceeded those expenditures by August 2020.^15^ The vast majority of conventional SLT advertising expenditures were through print media (96%), whereas most ONP promotion spending was through TV (61%) and radio (23%). Between 2019 and 2021 VELO spent the most on advertising ($23.45 million), vastly exceeding the expenditures of the U.S. ONP market leader ZYN ($1.17 million).^11,81^ Ad occurrences and characteristics analyses showed that VELO was predominantly promoted through television (17%) and radio (79%), referring to “freedom,” “health claims,” “flavor,” and “innovation” in headlines, targeting designated geographic market areas. ON! was commonly promoted through online displays (98.5%), with ads focusing on “flavor,” prioritizing national distribution. ZYN was also commonly promoted through online displays (78%), with ad headlines emphasizing “freedom,” “health claims,” and “brand,” focusing on national distribution.^11^ The most common claims in direct-mail advertising for ON! and VELO (including lozenges) were that the product could be used anywhere (84%), was an alternative to other tobacco products (69%), and was tobacco-leaf free (55%).^82^

#### Retail Availability

An audit of tobacco retailers in four U.S. states in 2021 found that ONPs were available in nearly half of stores, but were more common in chain convenience stores and neighborhoods with more non-Hispanic White residents.^83^ An analysis of 2016–2020 Nielsen data (excluding e-commerce and tobacco specialty stores) found that convenience stores accounted for 97.7% of U.S. ONP sales.^80^

## Discussion

ONPs, mainly produced by major combustible tobacco manufacturers, have become widely available in the United States. While large variations in use prevalence estimates were observed across studies, nationally representative U.S. studies suggest current youth ONP use below 1.5% and lifetime use below 2.5% through 2023. Adult use estimates were largely limited to populations with a history of tobacco use and varied widely by age and tobacco/nicotine product use status. In view of recent sales trends, estimates may have increased in the past year.^84,85^ Industry advertising expenditures for ONPs have grown, exceeding those of traditional SLTs, suggesting increasing interest by tobacco companies in promoting ONPs.

The public health impact of ONPs will depend on the extent to which they replace or supplement the use of more harmful tobacco products, and contribute to the initiation of ONPs or other nicotine or tobacco product use among nicotine/tobacco-naïve populations. Thus, it will be essential to understand the demographics of ONP users and the patterns and timing of use and co-use with other products. While longitudinal data on ONP use patterns and transitions are missing due to the novelty of the product, our review of cross-sectional studies found that in populations with a history of tobacco/nicotine product use, higher lifetime ONP use was observed for younger adult males, current and lifetime SLT users, current smokers, and dual cigarette and ENDS users. Among U.S. adolescents, higher lifetime ONP use was observed in male and non-Hispanic White populations. Thus, ONPs seem to attract youth with characteristics similar to the lifetime adolescent SLT users.^32^ Studies of current ONP use commonly reported small sample sizes and limited predictive power of observed sociodemographic associations. In addition, measures of current use in the reviewed literature have been mainly limited to past 30-day use, and thus may not represent regular use. More accurate and consistent measurements of ONP use have been recommended.^86^ Continued monitoring is needed to understand the likelihood of supplemental ONP use, use as a replacement for combustibles and other tobacco products, and use in otherwise nicotine-naïve populations.

ONP products have the potential to help smokers and SLT users transition to a less harmful alternative. While scarce, non-industry-funded studies of the chemical composition of ONPs report a total of 180 chemicals and indicate the presence of some harmful and potentially harmful compounds, including TSNAs. Such compounds were found at much lower levels compared to cigarettes and SLT, with the exception of formaldehyde levels, comparable to or higher than SLT. We note the high variability of identified compounds and their levels across different ONP products. More generally, tobacco smoke contains over 7000 chemicals, including 69 identified as carcinogens,^87^ while SLTs contain nearly 4000 chemicals with 28 known carcinogens.^88,89^ However, comparing constituents per unit does not provide a meaningful conclusion about the toxicant levels to which the user is exposed. To compare levels of exposure between ONPs and other products, factors like nicotine content and user behavior need to be considered. For example, considering smokers who switch to ONPs, comparing the toxicant exposure from the amount of the ONP product with nicotine content equivalent to the cigarettes they smoked per day could provide more accurate estimates of differences in potential exposure. Since ONPs are not purported for inhalation, their potentially harmful constituents may be expected to impact oral, gastrointestinal, and cardiovascular health, rather than respiratory health.

Short-term in vitro toxicology studies, funded predominantly by industry, suggest substantially less ONP cytotoxicity than cigarettes. Wide variations were observed within industry studies and compared to non-industry-funded findings regarding cytotoxicity levels of ONPs relative to SLT. Industry-funded human studies suggest beneficial effects of ONPs in oral health after switching from SLT, and in levels of biomarkers of exposure and potential harm after short-term switching from cigarette to ONP use. Thus, findings suggest that ONPs may be substantially less toxic than cigarettes. Yet, given the past track record of tobacco-funded research bias,^90^ independent studies are urgently needed to confirm these findings. In particular, more independent chemical, toxicological, preclinical, and human studies are needed to determine the harm of ONPs relative to SLT, which in turn are less harmful than cigarettes.^91,92^ Also, toxicity will likely vary between ONP products. More research is needed to determine whether results from existing studies generalize to other products.

Another factor in the acceptance of ONPs as a potential alternative to cigarettes and SLT is the nicotine delivery and subjective rating of ONPs compared to cigarettes and other forms of smokeless. Pharmacokinetic studies by industry find that higher nicotine strength ONPs can deliver comparable or higher nicotine to the user than SLT or cigarettes, although with slower nicotine release than cigarettes. While ONPs generally demonstrated the ability to relieve nicotine withdrawal symptoms in smokers, consistently lower positive subjective ratings compared to cigarettes and SLT call into question their suitability as an effective substitute.

There is considerable concern in the tobacco control community about ONPs becoming a new form of nicotine dependence in the tobacco-naïve population, especially youth. While ONP use among U.S. adolescents remains relatively low, we found nearly 35%–42% ONP product awareness by adolescents and young adults, and 9%–21% of tobacco-naïve youth were susceptible to trying them. These findings suggest that ONP product appeal may reach others besides established tobacco users. Higher odds of ONP awareness and susceptibility to use were observed in ever and current combustible tobacco users, noncombustible tobacco users, and users of multiple tobacco products. In particular, higher awareness and susceptibility to use among dual cigarette and noncombustible product users may suggest a greater likelihood of situational than substitutional use of ONPs. Indeed, ONPs are often marketed as more discrete alternatives to combustibles, ENDS, or HTPs and cross-promoted to smokers as a “product that could be used anywhere.”^82^ Since they may be used where indoor smoking and ENDS/HTP use is prohibited, ONPs may encourage dual-use.^23,47,54,82^

Industry marketing strategies of ONPs include diverse demographic groups previously excluded from traditional smokeless product marketing (eg, females, people of color, and LGBTQ+).^93^ The wide range of ONP flavors may be particularly attractive to youth.^40,72,94^ Although tobacco companies claim to target smokers, they have also claimed that ONPs may increase the size of the nicotine product market, presumably by attracting youth and young adults who would not have otherwise smoked.^95,96^ By using ad headlines focusing on freedom, innovation, and flavor, and emphasizing that ONPs are “tobacco-free,” prominent ONP brands disassociate the product from more established tobacco products and may attract new tobacco-naïve users. In particular, the “tobacco-free” descriptor may confuse understanding of the source of nicotine: 17% of U.S. young adults wrongfully believed that tobacco-free ONPs contained neither tobacco nor nicotine.^97^ The present review found that a “tobacco-free” modified warning label was associated with reduced harm perceptions among young adults, which in turn were related to higher susceptibility to ONP use, particularly among non-tobacco users.

In addition to the potential implicit reduced harm claims used by the industry to market ONPs, and their similarity in appearance to NRT products,^98^ some tobacco companies manufacture both ONPs and other nicotine products, such as lozenges and gums, under the same brand name (eg, VELO and Rogue).^99,100^ These marketing tactics may confuse users, contributing to a blurring of the lines between cessation and recreational nicotine products.^98^ Moreover, misleading marketing claims of “flavor-ban approved,” while many U.S. states and localities have enacted restrictions on sales of flavored tobacco products, may further contribute to confusion about ONP regulation.^20,101^

In the United States, ONPs are currently neither authorized by the FDA for marketing as a modified-risk product, nor approved as a cessation product. If granted marketing authorization, industry marketing will need to be regulated. Standards for ONP content should be promulgated, particularly regarding nicotine levels, levels of toxic substances, and flavoring additives. FDA-mandated nicotine addiction warning labels should be enforced on product packaging without modifications. Globally, countries where ONPs are currently not regulated need to update their classification and regulatory policies to include ONPs, particularly those containing synthetic nicotine, which often fall outside of any regulatory purview.^11^

The results of this review should be interpreted in light of its limitations. Our literature search did not include gray literature databases and excluded articles published in languages other than English. This may have prevented us from identifying relevant studies, particularly from Scandinavian countries, where ONP use is particularly high.^102^ Furthermore, as a scoping review we did not set out to statistically synthesize data or critically appraise the included studies. As more ONP studies become available, focused systematic reviews will be needed to provide pooled estimates and judge the quality and certainty of the evidence. We did not pre-register our review protocol in any publicly available databases.

We identified several limitations of current ONP research. The U.S. adult prevalence data from the included surveys are not representative of the general population due to oversampling of current and former smokers and other tobacco users. The low prevalence of ONP users precluded some studies from conducting stratified analysis by race/ethnicity and socioeconomic status. The cross-sectional design of the surveys further limits the ability to analyze transitions from smoking and other tobacco product use to ONP use to estimate their net public health benefit. Longitudinal data on transitions to and from ONP use is needed to inform independent simulation modeling studies to adequately estimate public health impacts. At present, the only such study is by industry.^103^ Nationally representative surveys should incorporate consistent measurements of ONP use. Given the novelty of ONPs and their resemblance to other oral tobacco/nicotine products, surveys need to employ clear definitions of ONPs to avoid product misclassification and ensure accurate estimates.

## Conclusions

Our scoping review provides an initial summary of the current evidence on ONP use and the potential impact of ONPs on public health. Based in part on the evidence from industry-funded studies, ONPs appear to be less toxic than cigarettes, and may deliver comparable nicotine to smokers, providing a potentially less harmful alternative to combustible products. More studies are needed to determine the harm of ONPs relative to SLT. Rather than, or in addition to, increased cessation from more harmful products, industry marketing might encourage the initiation of ONPs by youth and situational and dual-use by adults. Future studies should assess the awareness of, susceptibility to, and initiation of ONPs in a population with no history of tobacco/nicotine use, and better understand patterns of regular use among users of other nicotine and tobacco products, including transition patterns. As ONPs evolve, independently funded research is needed to understand and update use patterns as well as toxicology and health effects of ONPs compared to combustible and noncombustible products and to nonuse.

## Supplementary material

Supplementary material is available at *Nicotine and Tobacco Research* online.

ntae131_suppl_Supplementary_Table_S1

ntae131_suppl_Supplementary_Table_S2

ntae131_suppl_Supplementary_Table_S3

ntae131_suppl_Supplementary_Table_S4

ntae131_suppl_Supplementary_Table_S5

ntae131_suppl_Supplementary_File_S1
