# Supplementary material for: The Potential Impact of Oral Nicotine Pouches on Public Health: A Scoping Review
Source: Nicotine Tob Res. 2024 Jun 17;27(4):598–610. doi: 10.1093/ntr/ntae131 (PMC11931220; doi:10.1093/ntr/ntae131)
Supplement: ntae131_suppl_Supplementary_Table_S1 [file ntae131_suppl_supplementary_table_s1.docx]

The potential impact of oral nicotine pouches on public health: A scoping review.

Nargiz Travis, Kenneth E. Warner, Maciej L. Goniewicz, Hayoung Oh, Radhika Ranganathan, Rafael Meza, Jamie Hartmann-Boyce, David T. Levy

Supplementary Table S1. Characteristics of the included studies.

|  | First author, year | Country | Funding | Outcome Category | Study Design | Population |
| --- | --- | --- | --- | --- | --- | --- |
| 1 | Keller-Hamilton et al., 2023^1^ | US (Ohio) | Federal | Beliefs and perceptions; Product characteristics | Randomized trial | Adult smokers ages ≥21 years (n=30) |
| 2 | Birdsey et al., 2023^2^ | US | Federal | Use patterns | Cross-sectional survey | Middle and high school students (n=22,069) |
| 3 | Dowd et al., 2023^3^ | US | Federal | Use patterns | Cross-sectional survey | Adult current ONP users ages ≥18 years (n=118) |
| 4 | Long et al., 2023^4^ | US (Ohio) | Federal | Beliefs and perceptions | Qualitative descriptive study (focus groups) | Adult smokers (n=19) and SLT users ages ≥21 years (n=18) |
| 5 | Morean et al., 2023^5^ | US | Federal | Beliefs and perceptions | Cross-sectional survey | Participants, ages 13+, oversampled current tobacco users (n=1,000) |
| 6 | Rinaldi et al., 2023^6^ | Germany | Federal | Toxicity | In-vitro | n.a. |
| 7 | Schneller et al., 2023^7^ | US | Federal | Use patterns | Cross-sectional survey | Adolescents, ages 16-19 |
| 8 | Vogel et al., 2023^8^ | US | Federal | Beliefs and perceptions | Randomized control trial | Young adults past-month tobacco/nicotine users ages 21–34 years and no intention to quit (n=37) |
| 9 | Azzopardi et al., 2023^9^ | Sweden/  Denmark | BAT | Toxicity | Cross-sectional clinical study | Adult exclusive users of ONPs, and current (≥10 cigarettes/day), former and never smokers ages 19-55 (n=195) |
| 10 | Back et al., 2023^10^ | Sweden/  Canada | Swedish Match | Toxicity | Chemical Analysis | n.a. |
| 11 | Couch et al., 2023^11^ | US | Federal | Use patterns | Cross-sectional survey | Adolescents and young adult ENDS users ages 14-20 (n=2,253) |
| 12 | Gaiha et al., 2023^12^ | US | Federal | Use patterns | Cross-sectional survey | Adolescents, young adults, and adults ages 13–40 (n = 6,131) |
| 13 | Keller-Hamilton et al., 2023^13^ | US (Ohio) | Federal | Beliefs and perceptions | Randomized trial | Young adult men ages ≥18 years (n=239) |
| 14 | Kramer et al., 2023^14^ | US | Federal | Use patterns; Beliefs and perceptions | Cross-sectional survey | Middle and high school students (n = 20, 413) |
| 15 | Ling et al., 2023^15^ | US | Federal | Marketing and sales | Content analysis | n.a. |
| 16 | Mallock-Ohnesorg et al., 2023^16^ | Germany | Federal | Toxicity | Chemical Analysis | n.a. |
| 17 | Mays et al., 2023^17^ | US (Ohio) | University/  Federal | Beliefs and perceptions | Cross-sectional experimental | Adult cigarette smokers,  ST (chew, snuff, dip, and snus) users, and non-users of cigarettes or ST ages ≥ 21(n=301) |
| 18 | Morean et al., 2023^18^ | US | Federal | Use patterns; Beliefs and perceptions | Cross-sectional survey | Young adults ages 18–25, oversampled current tobacco users (n=609) |
| 19 | Patel et al., 2023^19^ | US | Truth Initiative | Use patterns | Cross-sectional survey | Youth and young adults ages 15-24 (n = 7,832) |
| 20 | Rensch et al., 2023^20^ | US | Altria | Toxicity | Open-label, randomized, controlled, in-clinic, 5-parallel-group study | Adult smokers ages 21-65 (n = 144) |
| 21 | Sparrock et al., 2023^21^ | US | Federal | Use patterns; Beliefs and perceptions | Cross-sectional survey | Adult current and former tobacco users ages ≥ 21 (n=1,583). |
| 22 | Tackett et al., 2023^22^ | US, Southern California | Federal | Beliefs and perceptions | Cross-sectional survey | Adolescent (9^th^ and 10^th^ grade) never tobacco or nicotine users (n=1,289) |
| 23 | Tosakoon et al., 2023^23^ | US | Federal | Use patterns; Beliefs and perceptions | Cross-sectional survey | Youth and young adults ages 18–34, oversampled cigarette/e-cigarette users (n=942). |
| 24 | Vogel et al., 2023^24^ | US, Southern California | Federal | Beliefs and perceptions | Cross-sectional survey | Ninth- and tenth-grade student never tobacco users (n=3,129) |
| 25 | Emery et al, 2022^25^ | US | Federal | Marketing and Sales | Marketing surveillance data analysis | n.a. |
| 26 | Felicione et al., 2022^26^ | US | Federal | Use patterns; Beliefs and perceptions | Cross-sectional survey | Adult current and former smokers and e-cigarette users ages ≥18 (n=2,507) |
| 27 | Duan et al, 2022^27^ | US | Federal | Marketing and sales | Marketing surveillance data analysis | n.a. |
| 28 | Tattan-Birch et al., 2022^28^ | Great Britain (England, Scotland, and Wales) | Government | Use patterns | Cross-sectional survey | Adults ages ≥ 18 (n=25,698) |
| 29 | Harlow et al., 2022^29^ | US, Southern California | Federal | Use patterns | Cross-sectional survey | 9^th^ and 10^th^ graders (n=3,516) |
| 30 | Morean et al., 2022^30^ | US | Federal | Use patterns; Beliefs and perceptions | Cross-sectional survey | Young adults ages  18-25, oversampled current e-cigarette and other tobacco product users (n=630) |
| 31 | Alizadehgharib et al., 2022^31^ | Sweden | Swedish Match | Toxicity | Clinical and in-vitro study | Healthy adult Swedish snus users ages ≥ 19 (n=60) |
| 32 | McEwan et al, 2022^32^ | Sweden | BAT | Product characteristics; Beliefs and perceptions | Randomized, controlled, crossover clinical study | Healthy adult current dual users of snus and combustible cigarettes ages 19-55 (n=35) |
| 33 | Chapman et al., 2022^33^ | Sweden | Imperial Brands | Product characteristics; Beliefs and perceptions | Randomized, controlled open-label, cross-over clinical study | Healthy adult users of traditional tobacco products  (cigarettes and Scandinavian snus) ages ≥ 19 (n=24) |
| 34 | Azzopardi et al., 2022^34^ | Canada | Imperial Tobacco Canada | Product characteristics; Beliefs and perceptions | Randomized crossover clinical study | Healthy adult smokers ages 15-55 (n=34). |
| 35 | Vogel et al., 2022^35^ | US, Southern California | Federal | Beliefs and perceptions | Cross-sectional survey | Young adult never users of ONPs ages 19-23 (n=1,167) |
| 36 | Liu et al., 2022^36^ | US | Altria | Product characteristics; Beliefs and perceptions | Randomized, partial single-blind, 7-way crossover design | Adult dual cigarette and moist SLT users ages 21-65 (n=30) |
| 37 | Gentzke et al, 2022^37^ | US | Federal | Use patterns | Cross-sectional survey | Middle and high school students (N=27.41 million) |
| 38 | Jablonski et al., 2022^38^ | US | Enthalpy Analytical | Toxicity | Chemical analysis | n.a. |
| 39 | Mallock et al., 2022^39^ | Germany | Federal | Product characteristics; Toxicity | Chemical analysis | n.a. |
| 40 | Shaikh et al., 2022^40^ | US | Federal | Toxicity | In-vitro study | n.a. |
| 41 | Shaikh et al., 2022^41^ | US | Federal | Toxicity | In-vitro study | n.a. |
| 42 | Rose et al., 2022^42^ | US (New Jersey, Kentucky, North Carolina, and New York) | Federal | Marketing and sales | Cross-sectional analysis | n.a. |
| 43 | Miller-Holt, 2022^43^ | Canada, US | Japan Tobacco Int. | Toxicity | In-vitro study | n.a. |
| 44 | Yu et al., 2022^44^ | US | Imperial Brands | Toxicity | In-vitro study | n.a. |
| 45 | Park-Lee et al., 2022^45^ | US | Federal | Use patterns | Cross-sectional survey | Middle and high school  students (N=2.51 million) |
| 46 | Majmundar et al., 2022^46^ | US | Federal | Marketing and sales | Sales data analysis | n.a. |
| 47 | Azzopardi et al., 2021^47^ | N/A | BAT | Toxicity | Chemical Analysis | n.a. |
| 48 | Stanfill et al., 2021^48^ | US | Federal | Product characteristics | Chemical Analysis | n.a. |
| 49 | Delnevo et al., 2021^49^ | US | Federal | Marketing and sales | Sales data analysis | n.a. |
| 50 | East et al., 2021^50^ | Canada, England, USA | Federal | Use patterns | Cross-sectional survey | Adolescents ages 16-19 (n=11,838) |
| 51 | Dalrymple et al., 2021^51^ | N/A | BAT | Toxicity | In-vitro study | n.a. |
| 52 | Rensch et al., 2021^52^ | N/A | Altria | Product characteristics; Beliefs and perceptions | Single-blind, randomized, 7-way crossover study | Healthy adult smokers ages 21-65 (n=42). |
| 53 | Brose et al, 2021^53^ | UK | Government | Use patterns; Beliefs and perceptions | Cross-sectional survey | Adult current or former smokers and/or ENDS users ages ≥18 years (n=3,883) |
| 54 | East et al., 2021^54^ | N/A | BAT | Toxicity | In-vitro study | n.a. |
| 55 | Marynak et al, 2021^55^ | US | Federal | Marketing and Sales | Sales data analysis | n.a. |
| 56 | Havermans et al., 2021^56^ | Netherlands | Government | Use patterns; Beliefs and perceptions | Cross-sectional survey | youth and adults ages 13 and over (n=5805) |
| 57 | Li et al., 2021^57^ | Australia, Canada, England, US | Government | Use patterns | Cross-sectional survey | Adult current cigarette smokers (n=9,112) and recent ex-smokers (n=1,184) ages ≥18 years |
| 58 | Hrywna et al., 2021^58^ | US | Federal | Use patterns; Beliefs and perceptions | Cross-sectional survey | Current adult smokers ages ≥18 years (n=1,018) |
| 59 | Czaplicki et al, 2021^59^ | US | Truth Initiative | Marketing and sales | Marketing surveillance data analysis | n.a. |
| 60 | Lunell et al., 2020^60^ | Sweden | Swedish Match | Product characteristics | An open, randomized, crossover, single-dose administration trial | Adult snus users >19 years (n=29) |
| 61 | Plurphanswat et al., 2020^61^ | US | Tobacco Industry | Use patterns; Beliefs and perceptions. | Cross-sectional survey | Adult current ZYN users ages ≥18 (n=1,266) |
| 62 | Bishop et al., 2020^62^ | N/A | BAT | Toxicity | In-vitro study | n.a. |

SLT= Smokeless Tobacco. ENDS= Electronic Nicotine Delivery System. BAT=British American Tobacco. N/A=not available; n.a.= not applicable.

**References:**

1. Keller-Hamilton B, Alalwan MA, Curran H, et al. Evaluating the effects of nicotine concentration on the appeal and nicotine delivery of oral nicotine pouches among rural and Appalachian adults who smoke cigarettes: A randomized cross-over study. *Addiction.* 2023.

2. Birdsey J, Cornelius M, Jamal A, et al. Tobacco Product Use Among U.S. Middle and High School Students - National Youth Tobacco Survey, 2023. *MMWR Morb Mortal Wkly Rep.* 2023;72(44):1173-1182.

3. Dowd AN, Thrul J, Czaplicki L, Kennedy RD, Moran MB, Spindle TR. A Cross-Sectional Survey on Oral Nicotine Pouches: Characterizing Use-Motives, Topography, Dependence Levels, and Adverse Events. *Nicotine Tob Res.* 2024;26(2):245-249.

4. Long L, Alalwan MA, Keller-Hamilton B, et al. Perceptions of oral nicotine pouches & their marketing among Ohio Appalachia smokers and smokeless tobacco users. *PLoS ONE.* 2023;18(10):e0293597.

5. Morean ME, Gueorguieva R, O'Malley S, Krishnan-Sarin S. Including the term 'tobacco-free nicotine' in the nicotine addiction warning label mandated by the US Food and Drug Administration alters risk perceptions and use intentions. *Tob Control.* 2023.

6. Rinaldi S, Pieper E, Schulz T, et al. Oral nicotine pouches with an aftertaste? Part 2: in vitro toxicity in human gingival fibroblasts. *Arch Toxicol.* 2023;97(9):2343-2356.

7. Schneller LM, Felicione NJ, Hammond D, Goniewicz ML, O'Connor RJ. Tobacco-Free Oral Nicotine Product Use Among Youth in the U.S., 2019-2021. *AJPM Focus.* 2023;2(1):100061.

8. Vogel EA, Tackett AP, Unger JB, et al. Effects of flavour and modified risk claims on nicotine pouch perceptions and use intentions among young adults who use inhalable nicotine and tobacco products: a randomised controlled trial. *Tob Control.* 2023.

9. Azzopardi D, Haswell LE, Frosina J, et al. Assessment of biomarkers of exposure and potential harm, and physiological and subjective health measures in exclusive users of nicotine pouches and current, former and never smokers. *Biomarkers.* 2023;28(1):118-129.

10. Back S, Masser AE, Rutqvist LE, Lindholm J. Harmful and potentially harmful constituents (HPHCs) in two novel nicotine pouch products in comparison with regular smokeless tobacco products and pharmaceutical nicotine replacement therapy products (NRTs). *BMC Chem.* 2023;17(1):9.

11. Couch ET, Halpern-Felsher B, Werts M, Chaffee BW. Use of Emerging and Conventional Oral Tobacco Among Adolescent and Young Adult E-Cigarette Users. *Subst Use Misuse.* 2023;58(2):283-288.

12. Gaiha SM, Lin C, Lempert LK, Halpern-Felsher B. Use, marketing, and appeal of oral nicotine products among adolescents, young adults, and adults. *Addict Behav.* 2023;140:107632.

13. Keller-Hamilton B, Curran H, Stevens EM, Zettler PJ, Mays D, Ferketich AK. Effects of "Tobacco Free" Language in Warning Labels on Perceptions of Electronic Cigarettes and Nicotine Pouches among Young Adult Men: A Randomized Trial. *Subst Use Misuse.* 2023;58(10):1302-1306.

14. Kramer RD, Park-Lee E, Marynak KL, Jones JT, Sawdey MD, Cullen KA. Nicotine Pouch Awareness and Use Among Youth, National Youth Tobacco Survey, 2021. *Nicotine Tob Res.* 2023.

15. Ling PM, Hrywna M, Talbot EM, Lewis MJ. Tobacco-Derived Nicotine Pouch Brands and Marketing Messages on Internet and Traditional Media: Content Analysis. *JMIR Form Res.* 2023;7:e39146.

16. Mallock-Ohnesorg N, Rinaldi S, Malke S, et al. Oral nicotine pouches with an aftertaste? Part 1: screening and initial toxicological assessment of flavorings and other ingredients. *Arch Toxicol.* 2023.

17. Mays D, Long L, Alalwan MA, et al. The Effects of Oral Nicotine Pouch Packaging Features on Adult Tobacco Users' and Non-Users' Product Perceptions. *Int J Environ Res Public Health.* 2023;20(4).

18. Morean ME, Bold KW, Davis DR, Kong G, Krishnan-Sarin S, Camenga DR. Awareness, susceptibility, and use of oral nicotine pouches and comparative risk perceptions with smokeless tobacco among young adults in the United States. *PLoS ONE.* 2023;18(1):e0281235.

19. Patel M, Kierstead EC, Kreslake J, Schillo BA. Patterns of oral nicotine pouch use among U.S. adolescents and young adults. *Prev Med Rep.* 2023;34:102239.

20. Rensch J, Edmiston J, Wang J, Jin X, Sarkar M. A Randomized, Controlled Study to Assess Changes in Biomarkers of Exposures Among Adults Who Smoke That Switch to Oral Nicotine Pouch Products Relative to Continuing Smoking or Stopping All Tobacco Use. *J Clin Pharmacol.* 2023.

21. Sparrock LS, Phan L, Chen-Sankey J, et al. Nicotine Pouch: Awareness, Beliefs, Use, and Susceptibility among Current Tobacco Users in the United States, 2021. *Int J Environ Res Public Health.* 2023;20(3).

22. Tackett AP, Wong M, Cho J, et al. Willingness to Use Commercial Nicotine Gums, Lozenges, and Gummies Among Nontobacco Using Adolescents in Southern California. *J Adolesc Health.* 2023;72(2):277-286.

23. Tosakoon S, Romm KF, Berg CJ. Nicotine pouch awareness, use and perceptions among young adults from six metropolitan statistical areas in the United States. *Tob Prev Cessat.* 2023;9:19.

24. Vogel EA, Barrington-Trimis JL, Harlow AF, et al. Prevalence of and disparities in adolescents' susceptibility to novel oral nicotine products marketed as "tobacco-free". *Prev Med.* 2023;166:107387.

25. Emery SL, Binns S, Carter CC, Rose SW, Kostygina G. Characterising advertising strategies and expenditures for conventional and newer smokeless tobacco products. *Tob Control.* 2022.

26. Felicione NJ, Schneller LM, Goniewicz ML, et al. Oral Nicotine Product Awareness and Use Among People Who Smoke and Vape in the U.S. *Am J Prev Med.* 2022;63(4):611-618.

27. Duan Z, Henriksen L, Vallone D, et al. Nicotine pouch marketing strategies in the USA: an analysis of Zyn, On! and Velo. *Tob Control.* 2022.

28. Tattan-Birch H, Jackson SE, Dockrell M, Brown J. Tobacco-free Nicotine Pouch Use in Great Britain: A Representative Population Survey 2020-2021. *Nicotine Tob Res.* 2022;24(9):1509-1512.

29. Harlow AF, Vogel EA, Tackett AP, et al. Adolescent Use of Flavored Non-Tobacco Oral Nicotine Products. *Pediatrics.* 2022;150(3).

30. Morean ME, Bold KW, Davis DR, Kong G, Krishnan-Sarin S, Camenga DR. "Tobacco-free" Nicotine Pouches: Risk Perceptions, Awareness, Susceptibility, and Use among Young Adults in the United States. *Nicotine Tob Res.* 2022.

31. Alizadehgharib S, Lehrkinder A, Alshabeeb A, Östberg AK, Lingström P. The effect of a non-tobacco-based nicotine pouch on mucosal lesions caused by Swedish smokeless tobacco (snus). *Eur J Oral Sci.* 2022;130(4):e12885.

32. McEwan M, Azzopardi D, Gale N, et al. A Randomised Study to Investigate the Nicotine Pharmacokinetics of Oral Nicotine Pouches and a Combustible Cigarette. *Eur J Drug Metab Pharmacokinet.* 2022;47(2):211-221.

33. Chapman F, McDermott S, Rudd K, et al. A randomised, open-label, cross-over clinical study to evaluate the pharmacokinetic, pharmacodynamic and safety and tolerability profiles of tobacco-free oral nicotine pouches relative to cigarettes. *Psychopharmacology (Berl).* 2022;239(9):2931-2943.

34. Azzopardi D, Ebajemito J, McEwan M, et al. A randomised study to assess the nicotine pharmacokinetics of an oral nicotine pouch and two nicotine replacement therapy products. *Sci Rep.* 2022;12(1):6949.

35. Vogel EA, Barrington-Trimis JL, Kechter A, et al. Differences in Young Adults' Perceptions of and Willingness to Use Nicotine Pouches by Tobacco Use Status. *IJERPH.* 2022;19(5).

36. Liu J, Rensch J, Wang J, et al. Nicotine pharmacokinetics and subjective responses after using nicotine pouches with different nicotine levels compared to combustible cigarettes and moist smokeless tobacco in adult tobacco users. *Psychopharmacology (Berl).* 2022;239(9):2863-2873.

37. Gentzke AS, Wang TW, Cornelius M, et al. Tobacco Product Use and Associated Factors Among Middle and High School Students - National Youth Tobacco Survey, United States, 2021. *MMWR Surveill Summ.* 2022;71(5):1-29.

38. Jablonski JJ, Cheetham AG, Martin AM. Market Survey of Modern Oral Nicotine Products: Determination of Select HPHCs and Comparison to Traditional Smokeless Tobacco Products. *SEPARATIONS.* 2022;9(3).

39. Mallock N, Schulz T, Malke S, Dreiack N, Laux P, Luch A. Levels of nicotine and tobacco-specific nitrosamines in oral nicotine pouches. *Tob Control.*

40. Shaikh S, Tung WC, Lucas J, Yogeswaran S, Li D, Rahman I. Flavor Classification/Categorization and Differential Toxicity of Oral Nicotine Pouches (ONPs) in Lung Epithelial Cells. *bioRxiv.* 2022.

41. Shaikh SB, Tung WC, Pang C, Lucas J, Li D, Rahman I. Flavor Classification/Categorization and Differential Toxicity of Oral Nicotine Pouches (ONPs) in Oral Gingival Epithelial Cells and Bronchial Epithelial Cells. *Toxics.* 2022;10(11).

42. Rose SW, Annabathula A, Westneat S, et al. Neighborhood distribution of availability of newer tobacco products: A four-site study, 2021. *medRxiv.* 2022.

43. Miller-Holt J, Baskerville-Abraham I, Sakimura M, Fukushima T, Puglisi A, Gafner J. In vitro evaluation of mutagenic, cytotoxic, genotoxic and oral irritation potential of nicotine pouch products. *Toxicology Reports.* 2022;9:1316-1324.

44. Yu F, Rudd K, Pour SJ, et al. Preclinical Assessment of Tobacco-Free Nicotine Pouches Demonstrates Reduced in Vitro Toxicity Compared with Tobacco Snus and Combustible Cigarette Smoke. *Applied In Vitro Toxicology.* 2022;8(1):24-35.

45. Park-Lee E, Ren C, Cooper M, Cornelius M, Jamal A, Cullen KA. Tobacco Product Use Among Middle and High School Students - United States, 2022. *MMWR Morb Mortal Wkly Rep.* 2022;71(45):1429-1435.

46. Majmundar A, Okitondo C, Xue A, Asare S, Bandi P, Nargis N. Nicotine Pouch Sales Trends in the US by Volume and Nicotine Concentration Levels From 2019 to 2022. *JAMA Netw Open.* 2022;5(11):e2242235.

47. Azzopardi D, Liu C, Murphy J. Chemical characterization of tobacco-free "modern" oral nicotine pouches and their position on the toxicant and risk continuums. *Drug Chem Toxicol.* 2022;45(5):2246-2254.

48. Stanfill S, Tran H, Tyx R, et al. Characterization of Total and Unprotonated (Free) Nicotine Content of Nicotine Pouch Products. *Nicotine Tob Res.* 2021;23(9):1590-1596.

49. Delnevo CD, Hrywna M, Lo EJM, Wackowski OA. Examining Market Trends in Smokeless Tobacco Sales in the United States: 2011-2019. *NICOTINE & TOBACCO RESEARCH.* 2021;23(8):1420-1424.

50. East KA, Reid JL, Rynard VL, Hammond D. Trends and Patterns of Tobacco and Nicotine Product Use Among Youth in Canada, England, and the United States From 2017 to 2019. *J Adolesc Health.* 2021;69(3):447-456.

51. Dalrymple A, Bean EJ, Badrock TC, et al. Enamel staining with e-cigarettes, tobacco heating products and modern oral nicotine products compared with cigarettes and snus: An in vitro study. *Am J Dent.* 2021;34(1):3-9.

52. Rensch J, Liu J, Wang J, Vansickel A, Edmiston J, Sarkar M. Nicotine pharmacokinetics and subjective response among adult smokers using different flavors of on!® nicotine pouches compared to combustible cigarettes. *Psychopharmacology (Berl).* 2021;238(11):3325-3334.

53. Brose LS, McDermott MS, McNeill A. Heated Tobacco Products and Nicotine Pouches: A Survey of People with Experience of Smoking and/or Vaping in the UK. *Int J Environ Res Public Health.* 2021;18(16).

54. East N, Bishop E, Breheny D, Gaca M, Thorne D. A screening approach for the evaluation of tobacco-free 'modern oral' nicotine products using Real Time Cell Analysis. *Toxicol Rep.* 2021;8:481-488.

55. Marynak KL, Wang X, Borowiecki M, et al. Nicotine Pouch Unit Sales in the US, 2016-2020. *Jama.* 2021;326(6):566-568.

56. Havermans A, Pennings JLA, Hegger I, et al. Awareness, use and perceptions of cigarillos, heated tobacco products and nicotine pouches: A survey among Dutch adolescents and adults. *Drug Alcohol Depend.* 2021;229(Pt B):109136.

57. Li L, Borland R, Cummings KM, et al. Patterns of Non-Cigarette Tobacco and Nicotine Use Among Current Cigarette Smokers and Recent Quitters: Findings From the 2020 ITC Four Country Smoking and Vaping Survey. *Nicotine Tob Res.* 2021;23(9):1611-1616.

58. Hrywna M, Gonsalves NJ, Delnevo CD, Wackowski OA. Nicotine pouch product awareness, interest and ever use among US adults who smoke, 2021. *Tob Control.* 2022.

59. Czaplicki L, Patel M, Rahman B, Yoon S, Schillo B, Rose SW. Oral nicotine marketing claims in direct-mail advertising. *Tob Control.* 2022;31(5):663-666.

60. Lunell E, Fagerström K, Hughes J, Pendrill R. Pharmacokinetic Comparison of a Novel Non-tobacco-Based Nicotine Pouch (ZYN) With Conventional, Tobacco-Based Swedish Snus and American Moist Snuff. *Nicotine Tob Res.* 2020;22(10):1757-1763.

61. Plurphanswat N, Hughes JR, Fagerström K, Rodu B. Initial Information on a Novel Nicotine Product. *Am J Addict.* 2020;29(4):279-286.

62. Bishop E, East N, Bozhilova S, et al. An approach for the extract generation and toxicological assessment of tobacco-free 'modern' oral nicotine pouches. *FOOD AND CHEMICAL TOXICOLOGY.* 2020;145.
