# Supplementary material for: The Potential Impact of Oral Nicotine Pouches on Public Health: A Scoping Review
Source: Nicotine Tob Res. 2024 Jun 17;27(4):598–610. doi: 10.1093/ntr/ntae131 (PMC11931220; doi:10.1093/ntr/ntae131)
Supplement: ntae131_suppl_Supplementary_Table_S2 [file ntae131_suppl_supplementary_table_s2.docx]

The potential impact of oral nicotine pouches on public health: A scoping review.

Nargiz Travis, Kenneth E. Warner, Maciej L. Goniewicz, Hayoung Oh, Radhika Ranganathan, Rafael Meza, Jamie Hartmann-Boyce, David T. Levy

Supplementary Table 2. Prevalence estimates of lifetime and current ONP use in adults and adolescents by survey year, country, and tobacco use status.

| Author (Survey Year) | Country | Study Design | Population | Tobacco use status | Lifetime ONP use, % | Current ONP use, % |
| --- | --- | --- | --- | --- | --- | --- |
| **Birdsey et al. (2023)** | US | Nationally representative school-based cross-sectional survey (NYTS 2023) | Middle and high school students (n=22,069) | Not specific- general population | 2.3 | 1.5 |
| **Park-Lee et al. (2022)** | US | Nationally representative school-based cross-sectional survey (NYTS 2022) | Middle and high school students (N=2.51 million) | Not specific- general population | 2.3 | 1.1 |
| Patel et al. (2021-2022) | US | National cross-sectional survey | Youth and young adults ages 15-24 (n = 7,832) | Not specific- general population | 16 | 12 |
| Gaiha et al. (2021) | US | Cross-sectional survey of a convenience sample | Adolescents, young adults, and adults. Ages 13–40  years (n = 6,131) | Not specific- general population | 17.9 | 10.5 |
| **Kramer et al. (2021)** | US | Nationally representative school-based cross-sectional survey (NYTS 2021) | Middle and high school students (n = 20, 413) | Not specific- general population | 1.9 | 0.8 |
| **Gentzke et al. (2021)** | US | Nationally representative school-based cross-sectional survey (NYTS 2021) | Middle and high school students (N=27.41 million) | Not specific- general population | 1.9 | 0.8 |
| Harlow et al. (2021) | US, Southern California | Cross-sectional school-based survey | 9^th^ and 10^th^ graders (n=3,516) | Not specific- general population | 0.6 | N/A |
| Schneller et al. (2019-2021) | US | Population-based cross-sectional survey (ITC Youth 2019, 2020,2021) | Adolescents ages 16-19, Waves 3-5 | Not specific- general population | 3.5  (Aug, 2019)  4.0  (Feb, 2020)  3.8  (Aug, 2020)  4.8  (Feb, 2021)  4.1  (Aug, 2021) | 1.5  (Aug, 2019)  1.8  (Feb, 2020)  1.7  (Aug, 2020)  2.0  (Feb, 2021)  2.0  (Aug, 2021) |
| **Tattan-Birch et al. (2020-2021)** | Great Britain (England, Scotland, and Wales) | Representative cross-sectional survey | Adults ages ≥18 years (n=25,698) | Not specific- general population | N/A | 0.32 |
| **Havermans et al. (2020)** | Netherlands | Nationally representative cross-sectional survey | Adolescents ages 13-17 (n=406) | Not specific- general population | 0.3 | 0 |
| **Havermans et al. (2020)** | Netherlands | Nationally representative cross-sectional survey | Adults ages ≥18 years (n=5,399) | Not specific- general population | 0.6 | 0.07 |
| East et al. (2019) | US | Population-based cross-sectional survey (ITC Youth 2019) | Adolescents ages 16-19 (n=11,838) | Not specific- general population | N/A | 1.5 |
| East et al. (2019) | Canada | Population-based cross-sectional survey (ITC Youth 2019) | Adolescents ages 16-19 (n=11,714) | Not specific- general population | N/A | <1.5 |
| East et al. (2019) | England | Population-based cross-sectional survey (ITC Youth 2019) | Adolescents ages 16-19 (n=11,070) | Not specific- general population | N/A | <1.5 |
| Tosakoon et al. (2022) | US (Atlanta, Boston, Minneapolis, Oklahoma City, San Diego, and Seattle). | Cross-sectional survey | Youth and young adults ages 18–34 (n=942) | Oversampling of cigarette/ENDS users | 9.8 | 2.2 |
| Morean et al. (2021) | US | Cross-sectional survey of a convenience sample | Young adults ages 18–25 (n=609) | Oversampling of current tobacco users | 10.3 | N/A |
| Morean et al. (2021)◊ | US | Cross-sectional survey of a convenience sample | Young adults ages  18-25 (n=630) | Oversampled current ENDS and other tobacco product users | 9.7 | N/A |
| Couch et al. (2021) | US | National cross-sectional survey | Adolescents and young adults ages 14-20 (n=2,253) | ENDS users | 29 | 11 |
| **Sparrock et al. (2021)** | US | Nationally representative cross-sectional survey | Adults ages ≥21 (n=1583). | Current and former tobacco users | 16.4 | 3 |
| **Hrywna et al. (2021)** | US | Nationally representative cross-sectional survey | Adults ages ≥18 years (n=1,018) | Current established smokers | 5.6 | N/A |
| Felicione et al. (2020) | US | Population-based cross-sectional survey (ITC 2020) | Adults ages ≥18 years (n=2,507) | Current and former smokers and ENDS users | 3 | 0.9 |
| Li et al. (2020) | England, Canada, Australia, US | Population-based cross-sectional survey (ITC 2020) | Adults ages ≥18 years (n=10,296) | Current and former smokers | N/A | (England)  0.9 (Canada)  0.8 (Australia)  0.8  (US) |
| **Brose et al. (2019)** | UK | Representative cross-sectional survey | Adults ages ≥18 years (n=3,883) | Current or former smokers and/or ENDS users | 4.4 | 2.7 |

Representative surveys are marked in bold. NYTS= National Youth Tobacco Survey. ITC= International Tobacco Control Policy Evaluation Project. ENDS=Electronic Nicotine Delivery Systems. ◊ Use of synthetic nicotine pouches (e.g., Niin, Fre, Rush and 2One brands). N/A= Not available.
