# Supplementary material for: The Potential Impact of Oral Nicotine Pouches on Public Health: A Scoping Review
Source: Nicotine Tob Res. 2024 Jun 17;27(4):598–610. doi: 10.1093/ntr/ntae131 (PMC11931220; doi:10.1093/ntr/ntae131)
Supplement: ntae131_suppl_Supplementary_Table_S3 [file ntae131_suppl_supplementary_table_s3.docx]

The potential impact of oral nicotine pouches on public health: A scoping review.

Nargiz Travis, Kenneth E. Warner, Maciej L. Goniewicz, Hayoung Oh, Radhika Ranganathan, Rafael Meza, Jamie Hartmann-Boyce, David T. Levy

Supplementary Table S3. Demographic Characteristics of Lifetime and Current ONP Users

| Author (Survey Year) | Country | Study Design | Population | Tobacco use status | Lifetime use | Current use |
| --- | --- | --- | --- | --- | --- | --- |
| **Birdsey et al. (2023)** | US | Nationally representative school-based cross-sectional survey (NYTS 2023) | Middle and high school students (n=22,069) | Not specific- general population | ↑Male  ↑White  ↑High school (vs. middle school) | ↑Male  ↑High school (vs. middle school) |
| **Park-Lee et al. (2022)** | US | Nationally representative school-based cross-sectional survey (NYTS 2022) | Middle and high school students (N=2.51 million) | Not specific- general population | ↑Male  ↑White  ↑High school (vs. middle school) | ↑Male  ↑High school (vs. middle school) |
| Patel et al. (2021-2022) | US | National cross-sectional survey | Youth and young adults ages 15-24 (n = 7,832) | Not specific- general population | **↑** Ages 21+ vs. younger  **↑** Male  **↑** Lower income | **↑** Ages 21+ vs. younger  **↑** Male  **↑** Lower income |
| **Kramer et al. (2021)** | US | Nationally representative school-based cross-sectional survey (NYTS 2021) | Middle and high school students (n = 20, 413) | Not specific- general population | ↑Male  ↑White  ↑High school (vs. middle school)  ↑Ever SLT use  ↑Current SLT use | ↑Male  ↑High school (vs. middle school)  ↑Current SLT use |
| **Schneller et al. (2019-2021)** | US | Nationally representative cross-sectional survey (ITC Youth 2019, 2020,2021) | Adolescents ages 16-19, Waves 3-5 | Not specific- general population | ↑Age18 vs. younger  ↑Male  ↑White  ↑Current SLT use  ↑Current ENDS use  ↑Current cigarette use | ↑Age18 vs. younger  ↑Male  ↑White  ↑Current SLT use  ↑Current ENDS use  ↑Current cigarette use |
| **Sparrock et al. (2021)** | US | Nationally representative cross-sectional survey | Adults ages ≥21 (n=1583). | Current and former tobacco users | **↑** Ages 18-30 vs. 61+ (AOR=4.17; 95% CI=1.78-9.78)  **↑**Ages 31-45 vs. 61+ (AOR=5.49; 95% CI=2.47, 12.20)  **↑**Current SLT use vs. non-use (AOR= 3.36; 95% CI=1.86-6.09) | **↑** Ages 18-30 vs. 61+  (AOR=54.72; 95% CI=6.24- 480.21)  **↑**Ages 31-45 vs. 61+ (AOR=58.76; 95% CI=7.52- 459.23)  **↑** Current ENDS use (AOR=3.23; 95% CI=1.07- 9.76)  **↑** Current cigarette use (AOR= 2.51; 95%CI=1.02-6.21)  **↑** Current SLT use (AOR= 8.35; 95% CI=2.64, 26.42) |
| Morean et al. (2021) | US | Cross-sectional survey of a convenience sample | Young adults ages 18–25 (n=609) | Oversampling of current tobacco users | **↑** Younger age (AOR=0.84; 95% CI=0.71-0.99)  **↑** Lifetime ENDS use (AOR=6.90; 95% CI=1.30-36.73)  **↑** Lifetime cigars/cigarillo use (AOR=2.88; 95% CI=1.27-6.55) **↑** Lifetime SLT use (AOR=16.45; 95% CI=7.68-25.24) | N/A |
| Morean et al. (2021) ◊ | US | Cross-sectional survey of a convenience sample | Young adults ages 18-25 (n=630) | Oversampled current ENDS and other tobacco product users | **↑** Male (AOR=2.97; 95% CI=1.00-8.81)  **↑** White (AOR=3.44; 95% CI=1.10-10.75)  **↑** Lifetime SLT use (AOR=6.19; 95% CI=2.16-17.69)  ˟ Lifetime ENDS, cigarette, cigar or hookah use. | N/A |
| **Hrywna et al. (2021)** | US | Nationally representative cross-sectional survey | Adults ages ≥18 years (n=1,018) | Current established smokers | **↑** Ages 18-44 vs. >=45 (AOR=2.91;95% CI:1.40-6.03)  **↑** Lifetime use of SLT (AOR=10.00;95%CI:4.02-24.88)  **↑** Previous quit attempts of smoking with traditional methods (e.g., NRTs)  (AOR=4.18;95%CI:1.85-9.48).  ↓ Higher vs. lower education (AOR=0.44;95%CI:0.20-0.98).  ˟ Sex  ˟ Ethnicity  ˟ Lifetime ENDS use | N/A |
| **Tattan-Birch et al. (2020-2021)** | Great Britain (England, Scotland, and Wales) | Representative cross-sectional survey | Adults ages ≥18 years (n=25,698) | Not specific- general population | N/A | **↑** Men (PR=4.55; 95% CI=2.27-9.09)  **↑** Ages 35-44 vs. 18-24 (PR=1.09; 95% CI=0.43-2.80)  **↑** Current smokers (PR=13.60; 95% CI=5.46-33.89)  **↑** Recent former smokers (PR=15.21; 95% CI=4.03-57.42)  **↑** Long-term (>1 year) former smokers (PR=3.71; 95% CI=1.36-10.15) vs. never smokers  **↑** Current ENDS (PR=10.59; 95% CI=5.74-19.52) and NRT (PR=9.75; 95% CI=4.64-20.49) users vs. non-users |
| Felicione et al. (2020) | US | Population-based cross-sectional survey (ITC 2020) | Adults ages ≥18 years (n=2,507) | Current and former smokers and ENDS users | **↑**Age 18-24 vs. >=55, (AOR=31.21; 95% CI=9.24-105.41)  **↑** Males (AOR=3.61; 95%CI=1.85-7.02)  **↑** Current smokeless tobacco users vs. non users (AOR=5.35; 95%CI=2.41-11.87)  **↓** Low and moderate education vs. high education  ˟ Ethnicity  ˟ Income | N/A |
| **Brose et al. (2019)** | UK | Representative cross-sectional survey | Adults ages ≥18 years (n=3,883) | Current or former smokers and/or ENDS users | **↑** Age <45  **↑** Male  **↑** Higher education  **↑** Current smoking and vaping | N/A |
| Plurphanswat et al. (2017-2018)§ | US | Cross-sectional survey | Adults ages ≥18 years (n=1,266) | Current ZYN users | N/A | Vast majority were:  men, white, with at least high school diploma with income more than $50 000, current SLT users and former tobacco users (mainly consisting of former dual cigarette‐SLT users) |

Representative surveys are marked in bold. §Data provided by Swedish Match. ◊ Use of synthetic nicotine pouches (e.g., Niin, Fre, Rush and 2One brands). SLT= Smokeless tobacco. ENDS= Electronic nicotine delivery systems. AOR= Adjusted odds ratio. NRT= Nicotine replacement therapy. ↑Positive associations with ONP use. ↓Negative association with ONP use. ˟ No association with ONP use. N/A= Not available.
