# Supplementary material for: The Potential Impact of Oral Nicotine Pouches on Public Health: A Scoping Review
Source: Nicotine Tob Res. 2024 Jun 17;27(4):598–610. doi: 10.1093/ntr/ntae131 (PMC11931220; doi:10.1093/ntr/ntae131)
Supplement: ntae131_suppl_Supplementary_Table_S4 [file ntae131_suppl_supplementary_table_s4.docx]

The potential impact of oral nicotine pouches on public health: A scoping review.

Nargiz Travis, Kenneth E. Warner, Maciej L. Goniewicz, Hayoung Oh, Radhika Ranganathan, Rafael Meza, Jamie Hartmann-Boyce, David T. Levy

Supplementary Table 4. Pharmacokinetic profiles of ONPs compared to other tobacco/nicotine products.

| **Author, year** | **Funding** | **Sample** | **Test Product** | **Reference Product 1** | **Reference Product 2** |
| --- | --- | --- | --- | --- | --- |
| Lunell et al., 2020 | Swedish Match | Adult snus users, (n=29) | ZYN 3 mg, 6 mg, 8 mg | 8 mg General Snus:  AUCinf higher than for ZYN 3 mg  AUCinf lower than for ZYN 6 mg  Cmax higher than for ZYN 3 mg  Cmax lower than for ZYN 6 mg  Tmax comparable to ZYN 3 mg and ZYN 6 mg  2 x 8 mg General Snus:  AUCinf higher than for ZYN 8 mg  Cmax higher than for ZYN 8 mg | 18 mg Longhorn moist snuff:  AUCinf, Cmax and Tmax not statistically different to ZYN 8 mg |
| Rensch et al., 2021 | Altria | Adult smokers (n=42) | Six flavors of ON! 4 mg | Own cigarette brand:  Higher Cmax and shorter Tmax compared to ONPs | N/A |
| Liu et al., 2022 | Altria | Adult dual cigarette and moist SLT users (n=30) | ON!1.5 mg, 2 mg, 3.5 mg, 4 mg, and 8 mg | Own cigarette brand:  Cmax higher than for 1.5 mg, 2 mg, 3.5 mg, and 4 mg ON!  AUC0-180 comparable to 3.5 mg and 4 mg ON!  Cmax and AUC0-180 lower than for 8 mg ON!  Tmax shorter than all ONPs | Moist SLT:  Cmax higher than for 1.5 mg, 2 mg, and 3.5 mg ON!  Cmax comparable to 4 mg ON!  AUC0-180 higher than for 1.5 mg, 2 mg, 3.5 mg, and 4 mg ON!  Cmax and AUC0-180 lower than for 8 mg ON!  Tmax comparable to all ONPs |
| McEwan et al., 2022 | BAT | Adult dual snus and cigarette users (n=35) | LYFT 10 mg | Combustible cigarette:  Cmax and AUC0–6h lower than for LYFT 10 mg  Tmax shorter than all ONPs | Other ONP brands (ZYN 10 mg, Nordic Spirit 9 mg, Skruf 8 mg, On! 6 mg):  Cmax and AUC0–6h lower for ZYN 10 mg and Skruf 8 mg than for LYFT 10 mg Cmax and AUC0–6h comparable for Nordic Spirit 9 mg, ON! 6 mg and LYFT 10 mg. |
| Chapman et al., 2022 | Imperial Brands | Adult tobacco users (n=24) | ZoneX 5.8 mg and 10.1 mg | Combustible cigarette:  AUCt and Cmax higher than for ONPs.  Tmax shorter than ONPs. | N/A |
| Azzopardi et al., 2022 | BAT | Adult smokers (n=36) | 4 mg ONPs (brand name n/a) | Nicorette® 4 mg nicotine gum:  AUCt, Cmax and Tmax lower than for ONP | Nicorette® 4 mg lozenge:  AUCt, Cmax and Tmax comparable to ONP |
| Keller-Hamilton et al., 2023 | Federal | Adult smokers (n=30) | ZYN 3 mg and 6 mg | Combustible cigarette:  Plasma nicotine levels at T30 lower than for ZYN 6mg and comparable to ZYN 3 mg. | N/A |

AUCinf - area under the plasma nicotine concentration-time curve from 0 to infinity. AUC0-180 - area under the plasma nicotine concentration-time curve from 0 to 180 minutes. AUC0-6h - area under the plasma nicotine concentration-time curve from 0 to 6 hours. AUCt area under the plasma concentration–time curve from time 0 to the time of the last sampling timepoint. Cmax- maximum observed plasma nicotine concentration. Tmax- time to maximum measured plasma nicotine concentration.
