# Supplementary material for: The Potential Impact of Oral Nicotine Pouches on Public Health: A Scoping Review
Source: Nicotine Tob Res. 2024 Jun 17;27(4):598–610. doi: 10.1093/ntr/ntae131 (PMC11931220; doi:10.1093/ntr/ntae131)
Supplement: ntae131_suppl_Supplementary_Table_S5 [file ntae131_suppl_supplementary_table_s5.docx]

The potential impact of oral nicotine pouches on public health: A scoping review.

Nargiz Travis, Kenneth E. Warner, Maciej L. Goniewicz, Hayoung Oh, Radhika Ranganathan, Rafael Meza, Jamie Hartmann-Boyce, David T. Levy

Supplementary Table 5. Highest detectable levels of common toxins in ONPs and reference products.

| Author | Funding | Product  (nicotine) | Chromium a) | Ammonia  b) | Nickel  c) | Formaldehyde  d) | NNN  e) | NNK  f) | NAB  g) | NAT  h) |
| --- | --- | --- | --- | --- | --- | --- | --- | --- | --- | --- |
| Back et al., 2023 | Swedish Match | ZYN dry (7.63 ± 0.21 mg), ZYN moist (12 mg) # | 0.16 μg/g | 66 μg/g | 0.067 μg/g | 10.3 μg/g | * | * | * | * |
|  |  | NRT gum (2 mg) | 0.850 μg/g | 4.5 μg/g | 0.243 μg/g | * | * | * | * | * |
|  |  | NRT Lozenge (2 mg) | * | * | 0.086 μg/g | * | * | * | * | * |
|  |  | Snus (8.13 ± 0.38) # | 0.52 μg/g | 847 μg/g | 0.817 μg/g | 1.6 μg/g | 0.44 μg/g | 0.19 μg/g | 0.02 μg/g | 0.31 μg/g |
|  |  | Pouched snuff (8.47 ± 0.12) # | 0.463 μg/g | 3,900 μg/g | 0.800 μg/g | 2.0 μg/g | 2.53 μg/g | 0.92 μg/g | 0.24μg/g | 2.43 μg/g |
| Mallock et al., 2022 | Federal | 44 ONPs (n/a) | - | - | - | - | 12.9 ng/pouch | 5.4 ng/pouch | 5.6 ng/pouch | 2.7 ng/pouch |
| Azzopardi et al., 2022 | BAT | 4 LYFT ONPs (n/a) | <50-80 ng/g | - | * | 1.13 μg/g | * | * | * | * |
|  |  | NRT gum (4 mg) | 743 ng/g | - | 223 ng/g | * | * | * | * | * |
|  |  | NRT Lozenge (4 mg) | <50-70 ng/g | - | 80 ng/g | * | * | * | * | * |
|  |  | Snus (n/a) | 1700 ng/g | - | 2000 ng/g | 1.5 μg/g | 640 ng/g | 200 ng/g | n.a. | n.a. |
| Jablonski et al., 2022 | Enthalpy Analytical | 21 ONPs  (4-8 mg) | 456 ng/g | - | 1115 ng/g | 14 μg/g | 39.4 ng/g | 13.7 ng/g | * | 18.5 ng/g |
|  |  | 4 SLT  (6.8-12 mg) | 401 ng/g | - | 972 ng/g | 3.64 μg/g | 2104 ng/g | 3380 ng/g | 274 ng/g | 4168 ng/g |

NNK = 4-(methylnitrosamino)-1-(3-pyridyl)-1-butanone; NNN =N’-nitrosonornicotine; NAB = N’-nitrosoanabasine; NAT = N’-nitrosoanatabine. #Measured total nicotine. *Below quantification levels. N.a.- not analyzed. Levels typically found in combustible cigarettes: a) vs. 1.1-1.7 ng/cigarette range of chromium levels in combustible cigarette smoke;^1^ b) vs. 4.14 -6.92 mg/cigarette range of ammonia levels in combustible cigarette smoke;^2^ c) vs. 3-57 ng/cigarette range of nickel in combustible cigarette smoke;^1^ d) vs. 2-50 μg/cigarette range of formaldehyde in combustible cigarette smoke;^1^ e) vs. 18-171 ng/cigarette range of NNN levels in combustible cigarette smoke;^3^ f) vs. 13-122 ng/cigarette range of NNK levels in combustible cigarette smoke;^3^ g) vs. 4-22 ng/cigarette range of NAB levels in combustible cigarette smoke;^3^ h) vs. 19-145 ng/cigarette range of NAT levels in combustible cigarette smoke.^3^

1. U.S. Department of Health and Human Services. *How Tobacco Smoke Causes Disease: The Biology and Behavioral Basis for Smoking-Attributable Disease* Atlanta (GA): Centers for Disease Control and Prevention;2010.

2. Inaba Y, Uchiyama S, Kunugita N. Spectrophotometric determination of ammonia levels in tobacco fillers of and sidestream smoke from different cigarette brands in Japan. *Environ Health Prev Med.* 2018;23(1):15.

3. Edwards SH, Rossiter LM, Taylor KM, et al. Tobacco-Specific Nitrosamines in the Tobacco and Mainstream Smoke of U.S. Commercial Cigarettes. *Chem Res Toxicol.* 2017;30(2):540-551.
