# Supplementary material for: The Potential Impact of Oral Nicotine Pouches on Public Health: A Scoping Review
Source: Nicotine Tob Res. 2024 Jun 17;27(4):598–610. doi: 10.1093/ntr/ntae131 (PMC11931220; doi:10.1093/ntr/ntae131)
Supplement: ntae131_suppl_Supplementary_File_S1 [file ntae131_suppl_supplementary_file_s1.docx]

The potential impact of oral nicotine pouches on public health: A scoping review.

Nargiz Travis, Kenneth E. Warner, Maciej L. Goniewicz, Hayoung Oh, Radhika Ranganathan, Rafael Meza, Jamie Hartmann-Boyce, David T. Levy

Supplementary File S1. Search strategy for the PubMed Database

| **PubMed** | | |
| --- | --- | --- |
| **Date Searched** | **Search String** | **Results** |
| January 1, 2016 through January 10, 2024 | (("VELO"[Title/Abstract] AND "pouch*"[Title/Abstract]) OR ("on"[Title/Abstract] AND "pouch*"[Title/Abstract]) OR "zyn"[Title/Abstract] OR ("nicotine"[Title/Abstract] AND "pouch*"[Title/Abstract]) OR ("tobacco-free"[Title/Abstract] AND "oral"[Title/Abstract] AND "pouch*"[Title/Abstract]) OR ("modern"[Title/Abstract] AND "oral"[Title/Abstract] AND "nicotine"[Title/Abstract] AND "product*"[Title/Abstract]) OR ("tobacco-free"[All Fields] AND ("lobeline"[MeSH Terms] OR "lobeline"[All Fields] OR "smokeless"[All Fields] OR ("tobacco, smokeless"[MeSH Terms] OR ("tobacco"[All Fields] AND "smokeless"[All Fields]) OR "smokeless tobacco"[All Fields] OR "snus"[All Fields]) OR ("tobacco, smokeless"[MeSH Terms] OR ("tobacco"[All Fields] AND "smokeless"[All Fields]) OR "smokeless tobacco"[All Fields] OR "snuff"[All Fields] OR "snuffs"[All Fields]))) OR ("nicotine-containing"[All Fields] AND "pouch*"[All Fields]) OR ("snus-like"[All Fields] AND "product*"[All Fields])) | 199 |
